# Single-cell transcriptome profiling of the immune space-time landscape reveals dendritic cell regulatory program in polymicrobial sepsis

**DOI:** 10.7150/thno.72760

**Published:** 2022-05-29

**Authors:** Ren-qi Yao, Zhi-xuan Li, Li-xue Wang, Yu-xuan Li, Li-yu Zheng, Ning Dong, Yao Wu, Zhao-fan Xia, Timothy R. Billiar, Chao Ren, Yong-ming Yao

**Affiliations:** 1 Translational Medicine Research Center, Medical Innovation Research Division and Fourth Medical Center of the Chinese PLA General Hospital, Beijing 100853, China.; 2 Department of Burn Surgery, the First Affiliated Hospital of Naval Medical University, Shanghai 200433, China.; 3 Research Unit of Key Techniques for Treatment of Burns and Combined Burns and Trauma Injury, Chinese Academy of Medical Sciences, Beijing 100730, China.; 4 Department of Surgery, University of Pittsburgh, Pittsburgh, PA 15213, USA.

**Keywords:** single-cell analysis, sepsis, immunosuppression, dendritic cells

## Abstract

**Rationale:** Evident immunosuppression has been commonly seen among septic patients, and it is demonstrated to be a major driver of morbidity. Nevertheless, a comprehensive view of the host immune response to sepsis is lacking as the majority of studies on immunosuppression have focused on a specific type of immune cells.

**Methods:** We applied multi-compartment, single-cell RNA sequencing (scRNA-seq) to dissect heterogeneity within immune cell subsets during sepsis progression on cecal ligation and puncture (CLP) mouse model. Flow cytometry and multiplex immunofluorescence tissue staining were adopted to identify the presence of 'mature DCs enriched in immunoregulatory molecules' (mregDC) upon septic challenge. To explore the function of mregDC, sorted mregDC were co-cultured with naïve CD4^+^ T cells. Intracellular signaling pathways that drove mregDC program were determined by integrating scRNA-seq and bulk-seq data, combined with inhibitory experiments.

**Results:** ScRNA-seq analysis revealed that sepsis induction was associated with substantial alterations and heterogeneity of canonical immune cell types, including T, B, natural killer (NK), and myeloid cells, across three immune-relevant tissue sites. We found a unique subcluster of conventional dendritic cells (cDCs) that was characterized by specific expression of maturation- and migration-related genes, along with upregulation of immunoregulatory molecules, corresponding to the previously described 'mregDCs' in cancer. Flow cytometry and *in stiu* immunofluorescence staining confirmed the presence of sepsis-induced mregDC at protein level. Functional experiments showed that sepsis-induced mregDCs potently activated naive CD4^+^ T cells, while promoted CD4^+^ T cell conversion to regulatory T cells. Further observations indicated that the mregDC program was initiated via TNFRSF-NF-κB- and IFNGR2-JAK-STAT3-dependent pathways within 24 h of septic challenge. Additionally, we confirmed the detection of mregDC in human sepsis using publicly available data from a recently published single-cell study of COVID-19 patients.

**Conclusions:** Our study generates a comprehensive single-cell immune landscape for polymicrobial sepsis, in which we identify the significant alterations and heterogeneity in immune cell subsets that take place during sepsis. Moreover, we find a conserved and potentially targetable immunoregulatory program within DCs that associates with hyperinflammation and organ dysfunction early following sepsis induction.

## Introduction

Sepsis is characterized by life-threatening organ dysfunction due to a dysregulated host response to infection based on the definition of Sepsis 3.0. In fact, sepsis remains the leading cause of mortality for patients admitted to intensive care units (ICUs) 1, 2. With its incidence increasing worldwide, sepsis results in immense economic and societal costs annually 3, 4. Currently, specific biomarkers and therapeutic agents effective in diagnosing and treating sepsis remain limited 5, 6. This can be attributed to the significant heterogeneity of populations and the lack of a clear understanding of the pathogenesis of the aberrant host immune response. A response paradoxically exhibits the simultaneous presence of proinflammatory and anti-inflammatory features manifesting a disturbed homeostasis 7, 8. The immunosuppression of sepsis appears to be a major driver of morbidity, leading investigators to seek novel immunotherapeutic strategies targeting sepsis-induced immune depression 9, 10. A comprehensive view of the host immune response to sepsis is lacking as the majority of studies on sepsis induced immune dysfunction have focused on a single immune cell type 7. Previous gene expression analyses in sepsis have been applied mostly to bulk populations of cells isolated from whole blood or tissues with cell heterogeneity based on cell surface markers 11-14. However, emerging evidence has confirmed that significant heterogeneity can be identified even within the identical immune cell types 15-17. Given that, bulk sequencing-based gene expression profiles are likely to aggregate transcripts across large, heterogenous cell populations, these studies are unlikely to reveal the full range of cell states.

Single-cell RNA sequencing (scRNA-seq) represents a powerful tool for deciphering the range of distinct cell subtypes and discovering previously unidentified cell types and states 18-21. Of note, scRNA-seq technologies have been increasingly applied to resolve immune cell heterogeneity and identify cell type-specific disease signatures in various immune-related disorders, including rheumatoid arthritis, systemic lupus erythematosus, type I diabetes, inflammatory bowel disease, and trauma 18, 22-25. To date, several studies have profiled the immune cell landscape of septic patients at single-cell resolution, in which peripheral blood mononuclear cells (PBMCs) derived from septic patients were subjected to scRNA-seq 26-28. However, the incorporated populations across these studies remained largely heterogenous regarding source of infection and severity of illness. Meanwhile, all the studies to date have focused on the transcriptomic changes within monocytes due to the dramatic changes that take place in these cells in septic patients 28. Moreover, based on integrative scRNA-seq analyses of publicly available datasets, two recently published studies uncovered a conserved transcriptome signature of monocytes, which shared between coronavirus disease 2019 (COVID-19) and septic patients 29, 30. Dendritic cells (DCs) are professional antigen-presenting cells (APCs) that play pivotal roles in initiating and regulating immune responses upon pathogen invasion 31, 32. In addition to the well-studied type 1 and type 2 conventional DCs (cDC1s and cDC2s), monocytes have been demonstrated to upregulate the expression of major histocompatibility complex class II (MHC-II) and CD11c together with downregulation of Ly6C under inflammatory states. These cells have been designated monocyte-derived DCs (moDCs) due to their antigen presenting capacity 33-35. Of note, scRNA-seq analyses have been broadly applied in decoding heterogeneity across DC subsets, and their associations with various human diseases. Notably, CD26^+^ inflammatory cDC2s were found to prime T cell-specific immunity in respiratory viral infection as well as allergy 33. Moreover, Janela et al. demonstrated that a dermal cDC1 subcluster played a pivotal role in mediating the activation and recruitment of neutrophils against cutaneous bacterial infections 36. Other than canonical DC subsets, a recent report also identified the infiltration of a previously unrecognized CD123^int^ BDCA-2^+^ DCs in skin wounds, with its involvement in sterile inflammation and wound healing 37. These data highlight the necessity and significance of scRNA-seq in investigating the relationship between DC subsets and pathogenesis of multiple human diseases. Nevertheless, a study that specifically addressed complexity and heterogeneity within cDCs subsets in sepsis is currently lacking.

Here, to profile a comprehensive immune landscape of sepsis, we applied scRNA-seq analysis in a widely studied experimental model of sepsis. Importantly, we incorporated elements of both time and sampling across multiple compartments using freshly isolated cells. This strategy permitted us to describe the broad and dynamic heterogeneity among T, B, natural killer (NK), and myeloid cells during sepsis. Strikingly, we identified a subpopulation of cDCs that was characterized by specific expression of maturation- and migration-related genes, along with upregulation of immunoregulatory molecules, corresponding to the previously described 'mature DCs enriched in immunoregulatory molecules' (mregDCs) in various cancer types 38. Our characterization of these cells extended into humans through a secondary analysis of scRNA-seq dataset obtained from a large-scale single-cell study of COVID-19 patients 39. The landscape data serves as a new resource for the study of sepsis and the discovery of the upregulation of mregDC opens a new avenue for research into the role of these cells in critical illness.

## Materials and Methods

### Mice

Wild type C57BL/6J mice were purchased form the Laboratory Animal Science of Chinese Academy of Medical Sciences (Beijing, China). *Batf3* knockout (KO) (*Batf3*^-/-^) mice and *IRF4* KO (*IRF4^-/-^*) mice were provided by Shanghai Model Biological Center (Shanghai, China). Male mice aged six- to eight-week-old were adopted for *in vivo* experiments, and they were housed under specific pathogen-free (SPF) conditions. All practices were carried out in line with the National Institutes of Health Guide for the Care and Use of Laboratory Animals and approved by the scientific Investigation Board of the Chinese PLA General Hospital (No. SYXK2020-0015), Beijing, China.

### Mouse model of polymicrobial sepsis

Experimental model of polymicrobial sepsis was established using cecal ligation and puncture (CLP) procedures 40. Male mice (N = 30) were anesthetized with 5% chloral hydrate (30 mg/kg) prior to disinfection of abdominal skin. One-centimeter-long median abdominal incision was performed to ensure adequately exposure of the cecum. Thereafter, cecum was ligated below the ileocecal valve and punctured once using 21-gauge needle followed by extradition of a fraction feces by compressing the ligated cecum. After relocating the cecum with close of incision, CLP mice were subsequently undergone a fluid resuscitation *via* subcutaneous injection of 0.9% normal saline. As for the sham group, mice solely underwent cecum exposure without performing CLP. To evaluate the successful reproduction of sepsis model, the occurrence of lethargy, diarrhea, and piloerection were observed after operations. At different intervals after CLP surgery (8, 24, and 72 h), mice were anesthetized using chloral hydrate, followed by collection of retro-orbital bleeding. After sacrificing the mice through euthanizing with CO_2_, various tissues were then harvested, including bone marrow, spleen, liver, heart, kidney, and lung.

### Isolation of murine immune cells

PBMCs were isolated using density gradient centrifugation based PBMCs isolation kit (TBDsciences, Tianjin, China) following the manufacturer's recommendations. In brief, whole blood (700-800 μL for each mouse) was obtained from murine retro-orbital bleeding using sodium heparin blood collection tubes and diluted with precooled phosphate-buffered saline (PBS) containing EDTA. Next, washed cell suspensions were superimposed on the surface of lymphoprep and centrifugated for 30 min at 450 g, followed by collection of interphase-containing PBMCs. Thereafter, red blood cells were lysed with lysis buffer for 5 min, which was terminated through adding RPMI supplemented with 2% fetal bovine serum (FBS). Cells were washed and resuspended using PBS containing bovine serum albumin (BSA) for further processing. To isolate bone marrow derived leukocytes, whole bone marrow cells were flushed from the femur with PBS containing 15 mM EDTA and 1% BSA. Suspensions were filtered by a 70 μm strainer, and cell debris was discarded by centrifugation for 10 min at 500 g. Complying the manufacturer's instructions for murine bone marrow lymphoprep (TBDsciences, Tianjin, China), re-suspended cells were layered onto the surface of lymphoprep resolution carefully, which were subsequently centrifugated for 30 min of 500 g at room temperature. Thereafter, bone marrow derived leukocytes were obtained by collecting the middle layer of the mixture. For splenic leukocyte isolation, spleens were harvested and dispersed in PBS, followed by disaggregation using a 70 μm cell strainer. After collecting splenocytes, leukocytes were derived using Ficoll-Paque density gradient centrifugation (500 g for 15 min). Following centrifugation, interphase-containing cells were collected, washed, and resuspended in PBS containing 1% BSA. Splenic leukocytes were collected as previously described, followed by isolation of splenic DCs using a CD11c^+^ DC isolation kit (MiltenyiBiotec, BergischGladbach, Germany) complying with the manufacturer's protocols.

### Single cell collection and single-cell RNA-seq

Following the manufacturer's guidelines, CD45^+^ immune cells were sorted using fluorescence-activated cell sorting (FACS) from the leukocytes stained with anti-mouse CD45 antibody. Thereafter, we used Countess II Automated Cell Counter to estimate viability and density of CD45^+^ cells stained with trypan blue, and single with viability higher than 85% was eligible for further sequencing. Cell suspensions were then processed for scRNA-seq using Chromium Controller. Library was prepared in accordance with the 10× genomics protocol for Single Cell 3' tag Gene Expression (10× Genomics, Pleasanton, CA). After performing single-cell gel bead-in-emulsions reverse transcription, PCR amplification was carried out to yield sufficient indexed complementary DNA (cDNA) for constructing scRNA-seq library. Finally, the libraries were sequenced on an Illumina HiSeq4000 (Illumina, San Diego, CA).

### Single-cell RNA-seq analysis

Cell Ranger Single-Cell Software Suite (10× Genomics, Pleasanton, CA) was adopted to convert Illumina base call files to FASTQ files with the 'cellranger mkfastq' function. It was subsequently used to align the sequenced reads to the reference genome (mm10 mouse transcriptome) and quantify expression level of transcripts for single-cell. Default quality control was performed to remove low-quality reads. This pipeline yielded files containing expression matrix among all samples, which documented unique molecular identifiers (UMIs) for each gene corresponding to each cell.

### Single-cell RNA-seq analysis-Data processing

Subsequent analyses were implemented using R software version 4.0.3 and the 'Seurat' package version 3.1.1 41. Cells with detected genes lower than 200 as well as mitochondrial content greater than 10% were filtered out during the quality control process. In line with gene expression signature, cells confirmed as doublets or multiplets were removed as well using the function 'DoubletFinder', when two or more population marker genes were highly expressed in single cell. Cells with UMI count above 40000 and detected genes above 5000 were also removed to exclude potential doublets. Following exclusion of low-quality cells, filtered UMI counts were normalized using the function 'NormalizeData', in which normalization method was designated as 'logNormalize' with a scaling factor of 10000.

### Dimension reduction and unsupervised clustering

'FindVariableGenes' function was performed with default parameters to select the highly variable genes, which were applied in conducting the linear dimensionality reduction. Next, principal component analysis (PCA) was performed regarding the top 2000 highly variable genes using 'RunPCA' function. Thereafter, numbers of PCs were selected corresponding to the ElbowPlot, followed by performing the 'RunUMAP' function with a perplexity value of 30 to acquire bidimensional coordinates for single-cell. In addition, we performed unsupervised cell clustering using 'FindClusters' function with a resolution of 0.6 on the basis of the identical PCs as for the 'RunUMAP' function. Consequently, the datasets were visualized by uniform manifold approximation and projection (UMAP) plots 42.

### Identification of differentially expressed genes (DEGs)

Based on the normalized data, DEGs across clusters were identified using function 'FindAllMarkers' or 'FindMarkers'. We used Bonferroni correction method to adjust for *P* values, and DEGs with adjusted *P* values higher than 0.05 were ruled out. Differential expression analysis between each immune cell subcluster was conducted using non-parametric Wilcoxon rank sum test from the 'Seurat' package.

### Cell cycle and pseudotime transcriptional trajectory analysis

Based on the expressions of marker genes associated with G2M or S phase, cells were designated a fraction of cell cycle. We applied 'CellCycleScoring' function to yield cell cycle score for each cell, which was subsequently matched into the metadata. This pipeline results in predicted classification for each cell in distinct proliferative phases was also calculated and grouped *via* the above package.

Trajectory and pseudotime analysis were carried out using 'Monocle2' package with the top 400 signature genes derived from the 'DifferentialGeneTest' function, which was designed to infer the potential developmental trajectory 43. The RNA counts among all cells from the subclusters were selected as input of 'Monocle2' for downstream analysis. Generalized additive models (GAMs) were constructed to generate the mean expression of each isoform. Tobit model was applied to access the relative gene expression of each cell. The linage differential trajectory among cDC subpopulations was performed using the default parameters of 'Monocle' following dimension reduction and cell ordering.

### Cell-cell interaction network analysis

To gain insight into the potential interactions between disparate immune cell types, we performed cell-cell network analysis to explore significant ligand-receptor pairs using 'CellPhoneDB' package 44. The interaction intensity between two immune cell subsets was determined using permutation test. Bonferroni multiple testing correction was applied for adjusting *P* value that was calculated across the ligand-receptor pairs. Ligand-receptor pairs with interacting intensity larger than 10 as well as an adjusted *P* value less than 0.01 were regarded as potential molecular partner that mediated cell-cell communications.

### Analysis of public scRNA-seq datasets

Publicly available scRNA-seq datasets were downloaded from Gene Expression Omnibus (GEO) as well as Single Cell Portal (SCP) using accession codes. GSE151658 scRNA-seq dataset containing renal cells isolated from LPS challenged mice was used to enrich murine DCs subset using R package 'Seurat' as mentioned above 45. Immune cells were re-clustered and annotated in line with our definition of cDCs (*Cd74*) and pDCs (*Siglech*). For the data of COVID-19 patients, we applied GSE158055 scRNA-seq dataset for subsequent analysis 39. BALF derived cells were exclusively incorporated, in which cluster information and gene expression from DCs were used as described in the article. SCP548 dataset containing CD45^+^ cells and DCs from PBMCs of septic patients was downloaded and used for secondary analysis. Clustering was carried out using human DCs marker genes (*CD1C* and *CLEC9A*), corresponding to DCs subtypes 28. To identify the presence of mregDCs, we subclustered DCs and profiled the expression level of mregDCs signature genes among DCs subpopulations.

### Bulk RNA sequencing and data processing

Total mRNA was extracted from splenic DCs sample by use of the Qiagen RNeasy Mini Kit (Qiagen, Mainz, Germany) complying for the manufacturer's instruction booklets. cDNA libraries were sequenced on an Illumina HiSeq2500 (Illumina, San Diego, CA). We assessed the quality of sequenced reads and filtered out reads of low quality by applying FastQC and MultiQC, followed by alignment to the mouse reference sequence (GRCm38/mm10). The counts were quantile normalized followed by log transformation, and they were then converted to expression matrix for downstream analysis.

### Histological examination

The dissected spleen, liver, lung, heart, and kidney were fixed in 4% paraformaldehyde (PFA) overnight at 4 °C, and embedded in paraffin blocks. Cryosections of the tissues (4-5 μm) were deparaffinized, followed by staining with hematoxylin-eosin (HE) for histological assessment. Histological manifestations were observed and analyzed *via* microscopy (Nikon Instruments Co., Japan). Two experienced histologists independently evaluated the sections, who were unaware of the grouping. The histological score of organs were calculated based on a four-point scale (0 [absent] to 3 [severe]) assigned to each criterion, for which at least three microscopic areas were examined to score each specimen (Table 1) 46, 47.

### Multiplex immunofluorescence tissue staining

Specimens of mouse spleen were stained using Opal Multiplex Immunohistochemistry Detection Kit (Akoya Biosciences, Marlborough, MA), and images were obtained with Vectra 3.0 Pathology Imaging System Microscope (Perkin-Elmer, Fremont, CA). Tissue sections of spleen were firstly deparaffinized, rehydrated, followed by antigen retrieved using microwave treatment. Subsequently, slides were blocked with 1% BSA containing 0.1% Triton X-100 (Sigma, St. Louis, MO) after keeping in 3% H_2_O_2_ for 15 min. Cryosections were incubated with primary antibodies, including anti-CD11c (Abcam, ab219799), anti-MHC-II (I-A/I-E) (Thermo Fisher Scientific, 14-5321-82), anti-CD80 (Abcam, ab215166), and anti-PD-L1 (Abcam, ab233482), and detection dye corresponding to each antibody was listed as follows: Opal520 dye (CD11c), Opal570 dye (MHC-II), Opal620 dye (PD-L1), and N700 dye (CD80). DAPI was adopted to counterstain nuclei. The digital images were analyzed using Halo Image Analysis software (Indica Labs, Corrales, NM).

### Flow cytometry

Flow cytometry were carried out using antibodies listed as follows: CD3e [BD Biosciences, 561824 (PE)], CD4 [Thermo Fisher Scientific, 11-0042-82 (FITC)], CD8a [BioLegend, 100706 (FITC)], CD11b [BD Biosciences, 561098 (PE/Cy7)], CD11c [BioLegend, 117353 (BV510)], CD19 [BD Biosciences, 561736 (PE)], CD25 [Thermo Fisher Scientific, 17-0251-82 (APC)], CD45 [BD Biosciences, 561037 (APC-Cy7)], CD49b [BD Biosciences, 561066 (PE)], CD80 [BioLegend, 104714 (APC)], CD274 [BioLegend, 124315 (BV421)], Ly-6G [BD Biosciences, 561104 (PE)], MHC-II (I-A/I-E) [BD Biosciences, 562363 (PerCP-Cy5.5)], and FOXP3 [BioLegend, 126419 (BV421)]. To determine the relative expression of surface markers, cells were stained in FACS buffer for 30 min at 4 °C. For intracellular staining, FOXP3 staining buffer kit (Thermo Fisher Scientific, Waltham, MA) was used following the manufacturer's instructions. Flow cytometry analyses were conducted on a LSR II instrument (BD Biosciences, Mountain View, CA). Results were obtained from the FACSDiva V 7.0 software (BD Biosciences, Mountain View, CA), and all data were analyzed by FlowJo Version 10.0 software. As for FACS, splenic mononuclear cells were isolated, prepared, and stained as described above, followed by sorting of mregDCs as well as non-mregDCs using BD FACSAria flow cytometer (BD Biosciences, Mountain View, CA).

### CD4^+^ T cell assay

Naïve CD4^+^ T cells were sorted from the spleen of untreated wild type C57BL/6J mice. 2 × 10^5^ CD4^+^ T cells were plated in complete RPMI 1640 medium, and stimulated with plate-bound anti-CD3 and anti-CD28 (1 μg/mL; Thermo Fisher Scientific, Waltham, MA) at a 1:1 ratio in 96-well plates for 24 h prior to subsequent experiments. 1 × 10^4^ mregDCs or non-mregDCs were subsequently added and co-cultured with T cells for another 72 h. Upon harvesting, co-cultural supernatants and CD4^+^ T cells were analyzed.

### Proliferation assay

To assess cell proliferation, we stained CD4^+^ T cells with CFSE Cell Division Tracker Kit (BioLegend, San Diego, CA) in accordance with the manufacturer's instructions. After activation and co-culture with sorted mregDCs or non-mregDCs, CD4^+^ T cells were harvested on day 4 and evaluated using flow cytometry. Cell proliferation was also quantified using CCK-8 reagent (Roche, Mannheim, Germany) in line with the manufacturer's specifications. The results were assayed by applying ELx808 absorbance readers (BioTek Instruments, Winooski, VT).

### Measurement of cytokine levels

Supernatants collected from co-culture medium were initially diluted for two-ten times with PBS + 1% BSA, and assayed by enzyme-linked immunosorbent assay (ELISA) kits (MyBioSource Inc., San Diego, CA) for measuring levels of IL-2, IL-4, IL-10, IL-12, IFN-γ, and high mobility group box-1 protein (HMGB1) according to the manufacturer's protocols. The results were analyzed by ELISA plate reader (Spectra MR, Dynex, Richfield, MN). Luminex liquid suspension chip detection was performed to quantify the level of various cytokines and chemokines in serum and splenic interstitial fluid derived from sham and CLP mouse. The Bio-Plex Pro Mouse Cytokine Grp I Panel 23-plex (Bio-Rad, Austin, TX) was applied in line with the manufacturer's instructions. The results were assessed using Bio-Plex MAGPIX System (Bio-Rad, Austin, TX).

### Western blotting

Protein extracts were loaded onto and separated by SDS-PAGE resolution (Pulilai Co., Beijing, China), followed by transferred onto a PVDF membrane. Next, membrane was blocked and incubated with primary antibodies and HRP-conjugated secondary antibody. Primary antibodies used in current experiments were listed: anti-NF-κB (Abcam, ab32536), anti-phospho-NF-κB (S536) (Abcam, ab76302), anti-IκBα (Cell Signaling Technology, 4812), anti-phospho-IκBα (S32) (Cell Signaling Technology, 2859), anti-TRAF2 (Cell Signaling Technology, 4712), anti-STAT3 (Cell Signaling Technology, 12640), anti-phospho-STAT3 (Y705) (Cell Signaling Technology, 9145), anti-IFNGR2 (ABclonal Technology, A7558), and anti-PD-L1 (Proteintech, 66248-1-Ig). β-actin was applied as a control for protein loading. Gels were visualized using the ECL system (Amersham Biosciences, Uppsala, Sweden) to determine the expression level of targeted proteins.

### Inhibitory experiment

To explore the role of NF-κB and STAT3 in mediating PD-L1 upregulation and mregDC program, wild type C57BL/6J mice (N = 40) were randomly yet equally divided into four groups: sham group, CLP group, CLP + JSH-23 group, and CLP + SH-4-54 group. DMSO dissolved JSH-23 (50 mg/kg) or SH-4-54 (10 mg/kg) was intraperitonially injected 30 min prior to CLP operation. JSH-23 and SH-4-54 were all purchased form Selleckchem (Houston, TX).

### Statistical analysis

All statistical analyses were carried out using R software (4.0.3) as well as GraphPad Prism software Version 8. All grouped data were summarized as mean ± standardized error mean (SEM). An unpaired student *t* test and one-way analysis of variance (ANOVA) were used to determine the statistical significance when two groups and more than three groups were compared, respectively. Linear regression analysis was carried out to testify the association between cell proportions and expression level of various cytokines as well as chemokines. Two-tailed *P* values less than 0.05 were deemed as having statistical significance.

## Results

### Single-cell profiling of the immune landscape in polymicrobial sepsis

**Figure 1:**
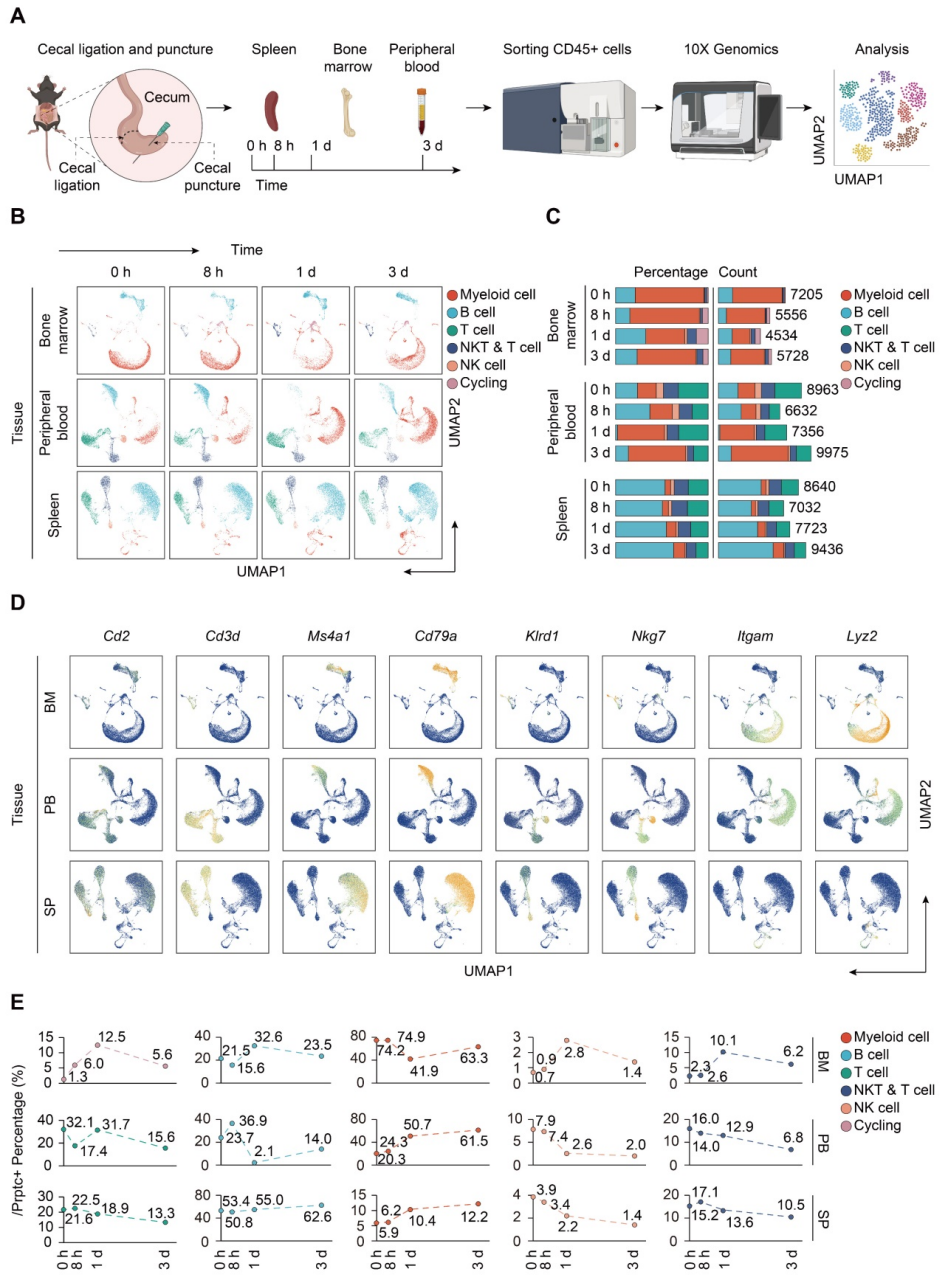
** Single-cell analyses reveal major immune cell composition in polymicrobial sepsis. A.** Schematic workflow depicting the experimental design of the current study. **B.** UMAP plots of major immune cell groups, across three immune-relevant tissue sites and time after cecal ligation and puncture (CLP) operation. **C.** Proportion and absolute counts of each major immune cell type in each sample. **D.** UMAPs showing expression of canonical annotation marker gene by color (blue, low expression; yellow, high expression). **E.** Proportion of major immune cell type at distinct time points among different tissue types, including bone marrow (BM), peripheral blood (PB), and spleen (SP).

To resolve the changes upon sepsis induction at the single cell resolution, we performed scRNA-seq of immune cells isolated from bone marrow, peripheral blood, and spleen from mice subjected to CLP, a widely accepted experimental model of sepsis 48. FACS-sorted CD45^+^ cells were subjected to 3′ tag scRNA-seq using the 10× Genomics Chromium platform. Overall, 88780 cells passing the quality control were eligible for subsequent analysis. A total of 21303 genes were identified, with median detected genes per cell higher than 1500. A schematic of the workflow was presented in Figure 1A. To visualize the qualitative changes in immune cell composition across tissue compartments and over time, cells were analyzed by the 'Seurat' package and displayed in 2-D space using UMAP (Figure 1B) 41. Cells from each tissue compartment and time point were shown as separate UMAP plots (colored by cell type, time 0 h = sham controls) and the percentages and absolute counts of all cell types within each sample were shown in Figure 1C. All major immune cell types were easily identified across collecting time points and sample types, albeit at different proportions. Based on the expression of canonical genes, the clusters were annotated into five major immune cell types: T cells (*Cd2*, *Cd3d*), B cells (*Ms4a1*, *Cd79a*), NK cells (*Klrd1*, *Nkg7*), myeloid-derived cells (*Itgam*, *Lyz2*), and cycling cells (Figure 1D; Table S1-S2) 49-51. The change in the relative abundance of each immune cell type over time was plotted across according to tissue types (Figure 1E; Table S3).

### Subtype analyses of immune cells during the course of sepsis

**Figure 2:**
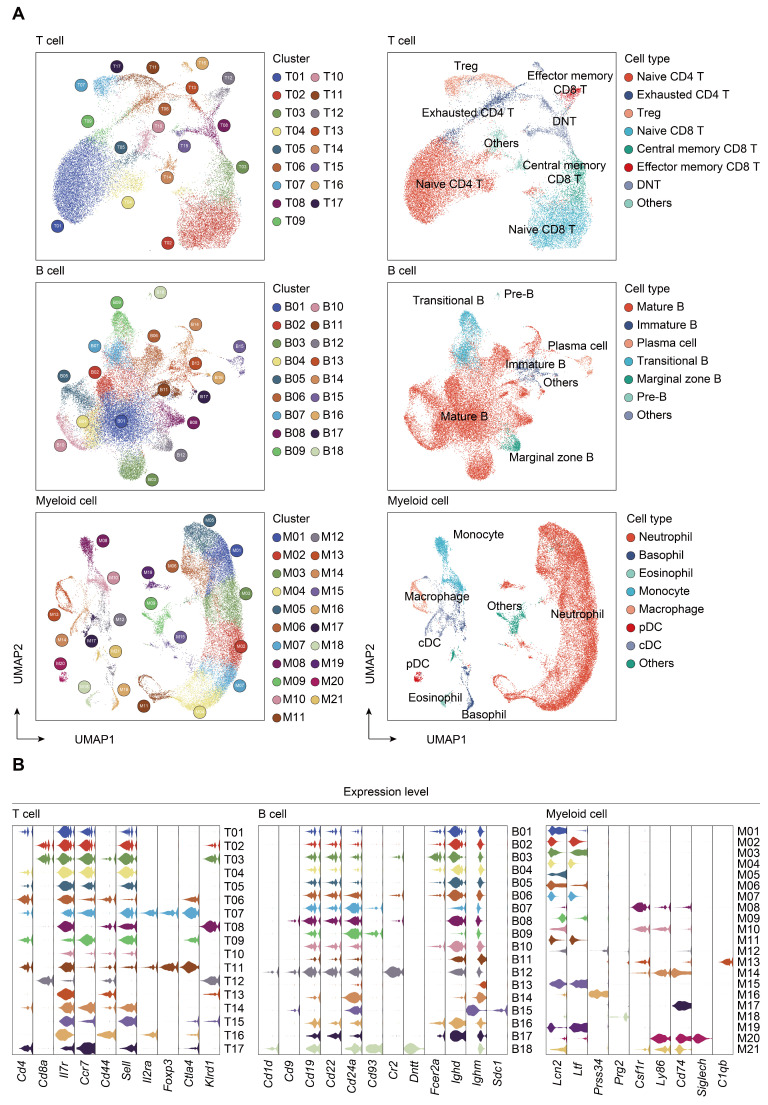
** Subtype analyses of T, B, and myeloid cells based on single-cell gene expression. A.** UMAP projections of subclustered T, B, and myeloid cells, labeled by distinguishing colors (left panels). Phenotypic annotations of each subcluster were presented in independent UMAP plots (right panels). **B.** Violin plots revealed expression level of selected marker genes for immune cell subsets within each lineage color coded by the subclusters shown in **A.**

**Figure 3:**
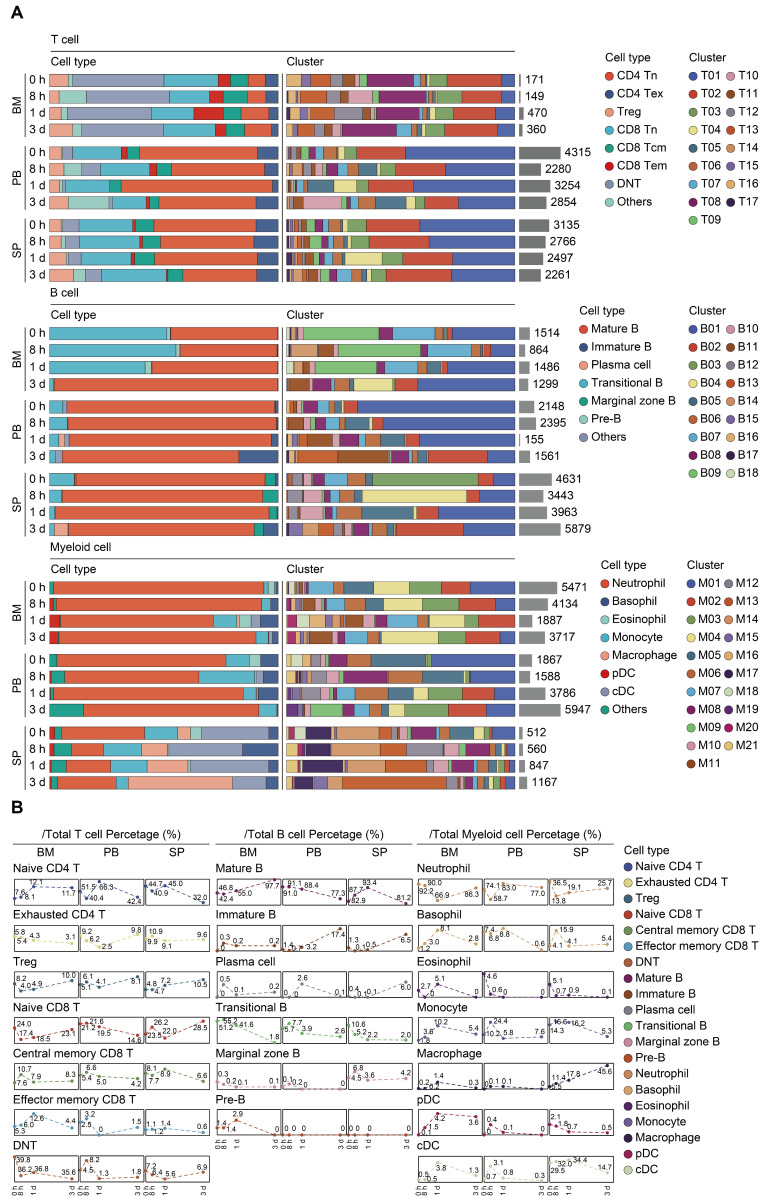
** Proportional and numerical alterations of T, B, and myeloid cells in sepsis. A.** Proportion and absolute counts of each subcluster of T, B, and myeloid cells, across three immune-relevant tissue sites and distinct time points after sepsis. **B.** Percentage of each annotated T/B/myeloid subtype at distinct time points across the anatomic sites.

Transcriptomic data from all cells across time and compartments were combined and unsupervised clustering analyses performed on each major immune cell type. A total of 17 subclusters emerged within the T lymphocytes, including 8 clusters for CD4^+^ cells, 3 clusters for CD8^+^ cells, and 4 clusters for double negative T cells (DNT) (Figure 2A-B; Table S4). T cells from four clusters (T01, T02, T04, and T05) revealed high expression of 'naïve' marker genes, including *Ir7r*, *Ccr7*, and *Sell* 52. Three CD4^+^ clusters (T06, T09, and T17) were characterized by the sole expression of *Ctla4*, in association with T cells with exhaustion phenotype. Cells from T07 and T11 expressed transcripts suggestive of regulatory T cells (Treg), which specifically expressed high level of *Foxp3* and *Il2ra* (*Cd25*) with low or intermediated expression of *Ir7r*. The second CD8^+^ cluster (T03) exhibited high expression of *Ccr7* and* Cd44*, commonly related to central memory T phenotype (Tcm). Additionally, cells from the T12 cluster expressed transcripts indicative of effector memory T cells (Tem), with high expression of *Cd44* and *Klrd1* along with negative expression of *Ccr7* 53. Of note, the high representation of naïve CD4^+^ T cells was sustainable over 24 h post-sepsis and even transiently higher than that of the sham group at 24 h after CLP, whereas splenic and circulating naïve CD4^+^ T cells underwent a substantial reduction at 72 h after sepsis. Meanwhile, the proportion of splenic *Cd4*^+^ *Cd25*^+^ *Foxp3*^+^ Treg continually increased after the onset of sepsis, and proportion of circulating Treg attained the highest level at 72 h (Figure 3A-B; Table S5).

A similar UMAP analysis was performed on B lymphocytes, yielding 18 subclusters based on differential expression of canonical genes (Figure 2A-B; Table S4) 54, 55. Eleven clusters (B01-B06, B08, B10, B11, B14, and B17) expressed high level of *Cd19*,* Ighd*, and *Ighm*, which were annotated as mature B cells. Cells from B07 and B09 clusters expressed transcripts indicative of transitional B cells, whereas B12 cluster was characterized by specific expression of *Cd1d*, *Cd9*, commonly associated with the phenotype of marginal zone B (MZB) cells. Cells from B13 cluster expressed transcripts indicative of immature B cells, with low expression *Ighm* along with negative expression of *Fcer2a* (*Cd23*) and* Ighd*. Cells from B15 and B18 clusters specifically expressed *Sdc1* (*Cd138*) and* Dntt* (*Tdt*), corresponding to plasma cells and pre-B cells, respectively. The percentage of mature B cells persistently declined in peripheral blood, while spleen-derived mature B cells remarkably increased at 24 h following septic challenge. Notably, a substantial expansion of immature B cells in peripheral blood and spleen was observed at 24 h after CLP, hinting an impaired B cell maturation in sepsis (Figure 3A-B; Table S5).

Re-clustering of myeloid cells yielded 21 subpopulations, for which annotations of neutrophils (M01-M07, M11, M15, and M19) (*Lcn2*, *Ltf*), monocytes (M08 and M10) (*Csf1r*, *Ly86*), basophils (M12 and M16) (*Prss34*), macrophages (M13) (*C1qb*), cDCs (M14 and M17) (*Cd74*), eosinophils (M18) (*Prg2*), and pDCs (M20) (*Siglech*) were based on the expression of canonical genes (Figure 2A-B; Table S4) 21, 56. The proportion of splenic monocytes diminished at 72 h after sepsis, while a cluster of macrophages that preferentially enriched in spleen substantially expanded upon the induction of sepsis. A dramatic loss in splenic plasmacytoid and conventional populations of DCs was observed at 72 h after CLP, in agreement with previous findings (Figure 3A-B; Table S5) 10, 57. NK cells were comprised of 2 clusters for* Itgam*^+^*Cd27*^+^ NK cells (N01 and N05), 5 clusters for* Itgam*^+^*Cd27*^-^ NK cells (N02-N04, N06, and N09), and 1 cluster for *Itgam*^-^*Cd27*^+^ NK cells (N07), whereas cycling cells could be further categorized into multipotent progenitor (MPP) (P07), megakaryocyte-erythroid progenitor (MEP) (P09), granulocyte-monocyte progenitor (GMP) (P01, P04-P06, and P10), and common lymphoid progenitor (CLP) (P02 and P03) (Figure S1A-D; Table S4) 58, 59. The absolute number and proportion of each subcluster across separate samples were shown in Figure S1E-F and Table S5. Collectively, these results highlight a substantial alteration of the immune system following the onset of sepsis.

### Trajectory and cell-cell interaction analyses of immune cells in sepsis

**Figure 4:**
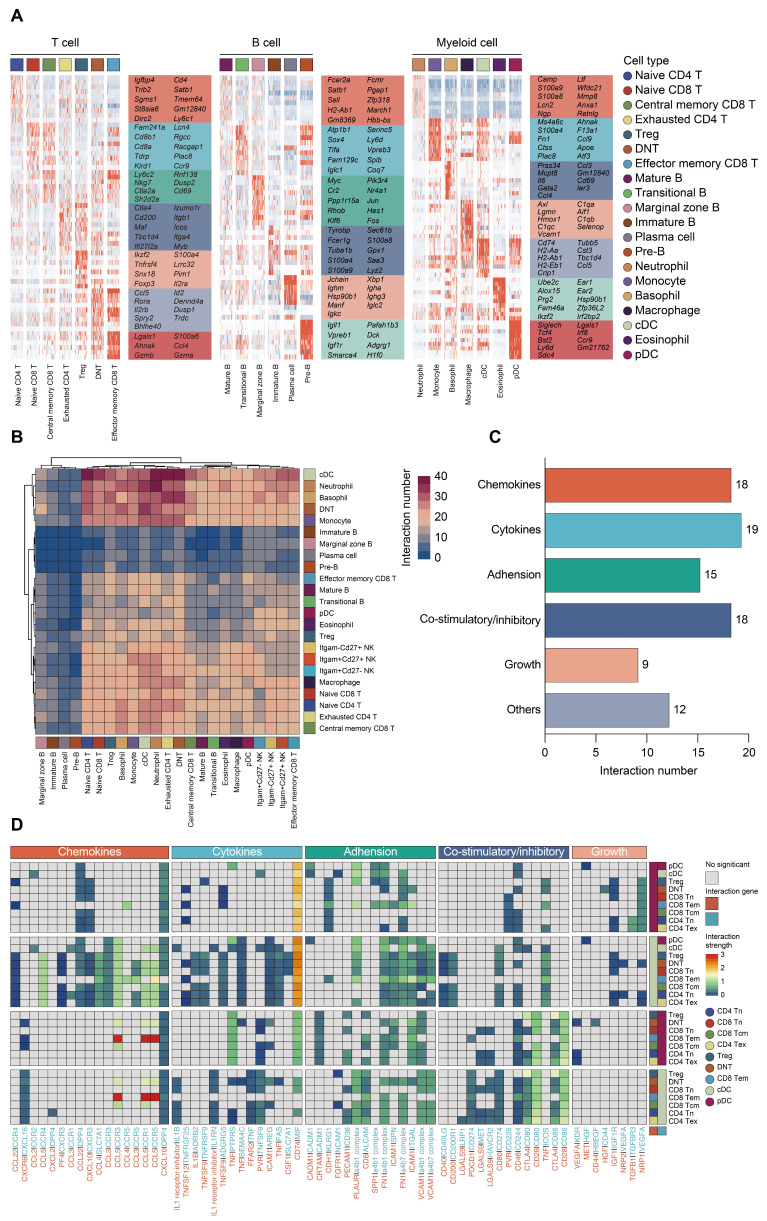
** Composition and cell-cell interacting network of the immune cells in sepsis. A.** Heatmaps displaying the relative expression level of cell type-specific genes across subclusters of T, B, and myeloid cells. **B.** Heatmap with double projection showing the cell-cell interacting density among all identified immune cell subtypes, which was proportional to the number of ligands when isogenic receptors were expressed in the recipient cell type (blue, low density of cell-cell interactions; purple, dense cell-cell interactions). **C.** Ligand-receptor pairs between T lymphocytes and DCs were categorized into six patterns in line with their biological functions, including chemokines, cytokines, adhesion, co-stimulatory/inhibitory, growth and others. Numbers of each pattern were ranked accordingly. **D.** Detailed analysis of the receptors expressed by each T lymphocyte and DC subtype and the cells expressing the cognate ligands primed to receive the signal. Statistical significance (*P*<0.05) was determined by permutation test using CellPhoneDB, with grey color indicating no statistical significance. The color gradient indicated the level of interaction (blue, low level of interaction; red, high level of interaction).

To confirm the annotation of each immune cell subset, we profiled the expression patterns of marker genes among subclusters based on scRNA-seq data (Figure 4A; Figure S1G; Table S6). To characterize the expression of transcripts for known ligand-receptor pairs and identify potential interactions between cell types, we analyzed the scRNA-seq data using the 'CellPhoneDB' package 60. Heatmap revealed dense intercellular communication among immune cells, especially between DCs and T lymphocytes (Figure 4B-C). Broadcast ligands for which isogenous receptors were identified indicated extensive communications between cDCs and T cells (Figure 4D). Notably, cDC subset exhibited expression of stimulatory molecules, including CD80, CD86 as well as CD40, and the paired receptors were widely expressed in all populations of T lymphocytes. Multiple immune checkpoint markers (CD274, HAVCR2, CD200) could be detected in cDCs as well, implying that various inhibitory communications were presented between cDCs and T lymphocytes. Thus, the phenotype of the DCs is likely to be a potential factor in the immune regulation of lymphocytes during sepsis (Figure 4C-D) 38, 61.

We next assessed the phenotypic alterations in immune cell populations in the development of sepsis. DCs had the lowest S phase and G2M phase score in comparison to other cell types, suggesting that DCs exerted low proliferative and self-renewal ability (Figure S2A-D). To gain insight into the temporal dynamics within immune cell clusters, we applied 'Monocle2' to conduct pseudotime and trajectory analyses on the basis of scRNA-seq data (Figure S3A) 43. The percent representation of cells from each cluster was shown by cell state. The putative trajectories based on cell states were summarized in Figure S3B-D. As expected, T cells had the largest number of states, but somewhat unexpected was the many differentiation states observed in NK cells during sepsis.

### Characterizing cDCs heterogeneity in sepsis

**Figure 5:**
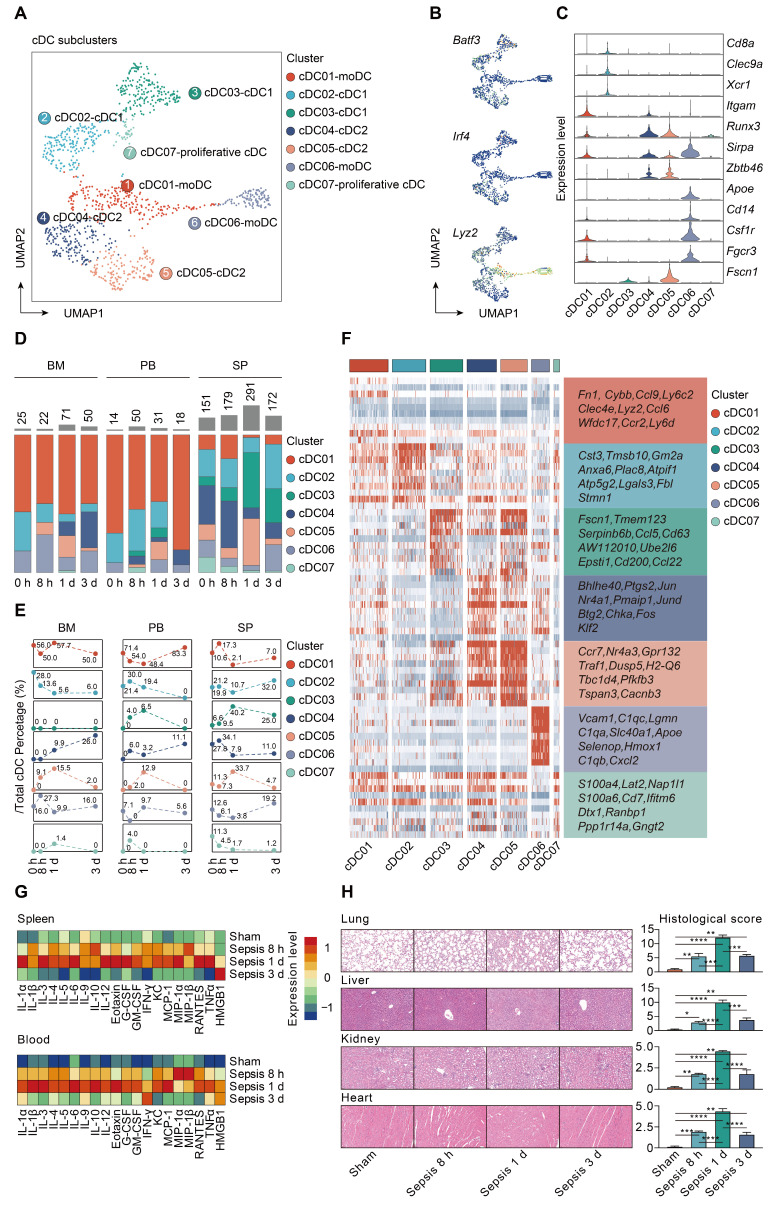
** Characterization of conventional dendritic cell subset in sepsis. A.** UMAP plot showing subclusters of conventional dendritic cells (cDCs) by color. **B.** UMAPs showing expression level of key lineage markers of cDCs, including *Batf3*, *Irf4* and *Lyz2*, corresponding to cDC1s, cDC2s and monocyte-derived DCs (MoDCs), respectively. **C.** Violin plots displaying expression level of marker genes for cDC subsets within each lineage (blue, low expression; yellow, high expression). **D.** Proportion and absolute counts of each subcluster of cDCs, across three immune-relevant tissue sites and distinct time points. **E.** Percentage of each cDC subclusters at distinct time points among different tissue types. **F.** Heatmaps displaying the relative expression level of top 10 differentially expressed genes (DEGs) among all cDC subpopulations. **G.** Heatmaps of Luminex liquid suspension chip analysis indicating relative expression level of various cytokines/chemokines in serum and splenic interstitial fluid derived from mice underwent sham or CLP surgery. **H.** Histological scores (right panel) and representative images of hematoxylin and eosin (HE) staining (left panel) elucidating the pathological alterations in multiple organs of mice underwent sham or CLP surgery, including lung, liver, kidney, and heart. One-way analysis of variance (ANOVA) with Tukey HSD test was applied to testify the statistical significance. Data were expressed as means ± standard error of mean (SEM). *P<0.05; **P<0.01; ***P<0.001; ****P<0.0001.

To gain insight into the heterogeneity in functional subtypes of cDCs in sepsis, an unsupervised re-clustering was performed on overall cDCs using a UMAP algorithm, yielding 7 subclusters (Figure 5A; Table S7). Murine cDCs could be further divided into cDC1s and cDC2s based on functional and developmental differences 16, 62. The development of cDC1s is driven by the transcription factors (TFs) BATF3 and interferon regulatory factor 8 (IRF8), while cDC2s are developmentally dependent on IRF4 63-65. Monocytes have been demonstrated to phenotypically shift to moDCs under inflammatory states 33. Herein, the identified populations were comprised of three major subtypes based on cluster-specific enrichment of *Batf3*, *Irf4*, and *Lyz2*, including 2 cDC1s clusters (*Cd8a*, *Clec9a*, *Xcr1*), 2 cDC2s clusters (*Itgam*, *Runx3*, *Sirpa*,* Zbtb46*), and 2 moDCs clusters (*Apoe*, *Cd14*, *Csf1r*, *Fgcr3*) (Figure 5B-C). Although cDC03 cluster did not express canonical cDC1s genes, it specifically expressed *Fscn1*, a cell migration marker, thus representing migratory cDC1s. Of note, cells from cDC05 cluster shared common genes with those of cDC04, and expressed high level of *Fscn1*, suggesting that these cells had a migratory cDC2s phenotype. In addition, cells from cDC07 cluster were identified as the proliferative cDCs, which were confirmed by the enrichment of both ribosome- and cell cycle-related genes (Figure 5B-C) 66.

moDCs were prevalent in bone marrow and peripheral blood, especially for the cDC01 cluster, while cDC1s and cDC2s were preferentially localized in spleen. The proportion of splenic derived non-migratory cDC1s and cDC2s underwent a substantial reduction in prevalence at 24 h following septic challenge. Conversely, cells forming the cDC05 cluster, while scarce in the sham group, were predominantly enriched at all time points after CLP, and could be observed among all tissue types. Since cDC05 cluster was provisionally annotated as migratory cDC2s, these results suggested a potential phenotypic shift from non-migratory subtype to populations bearing migratory capacity (Figure 5D-E; Table S8). Additionally, we profiled the expression level of the top 10 marker genes for each cDC subpopulation (Figure 5F; Table S9). *Ccr7* and *H2-Q6* (*MHC-II*), commonly associated with cell migration and antigen presentation, respectively, were upregulated in the cDC05 cluster. These findings indicate remarkable shifts in cDC populations that are time and tissue specific.

### Identification and confirmation of mregDCs upon sepsis induction

Our analyses showed that increased percentage of cDC05 cluster was significantly associated with elevated expression level of various cytokines and hyperinflammatory state under septic challenge, as evidenced by bio-plex and linear regression analyses (Figure 5G; Figure S4). Meanwhile, the emergence of the cDC05 cluster at 24 h after sepsis was correlated with injuries of multiple organs, which was determined by histological examination using standardized scoring system (Figure 5H). Consequently, we focused our subsequent analysis on cDC05.

**Figure 6:**
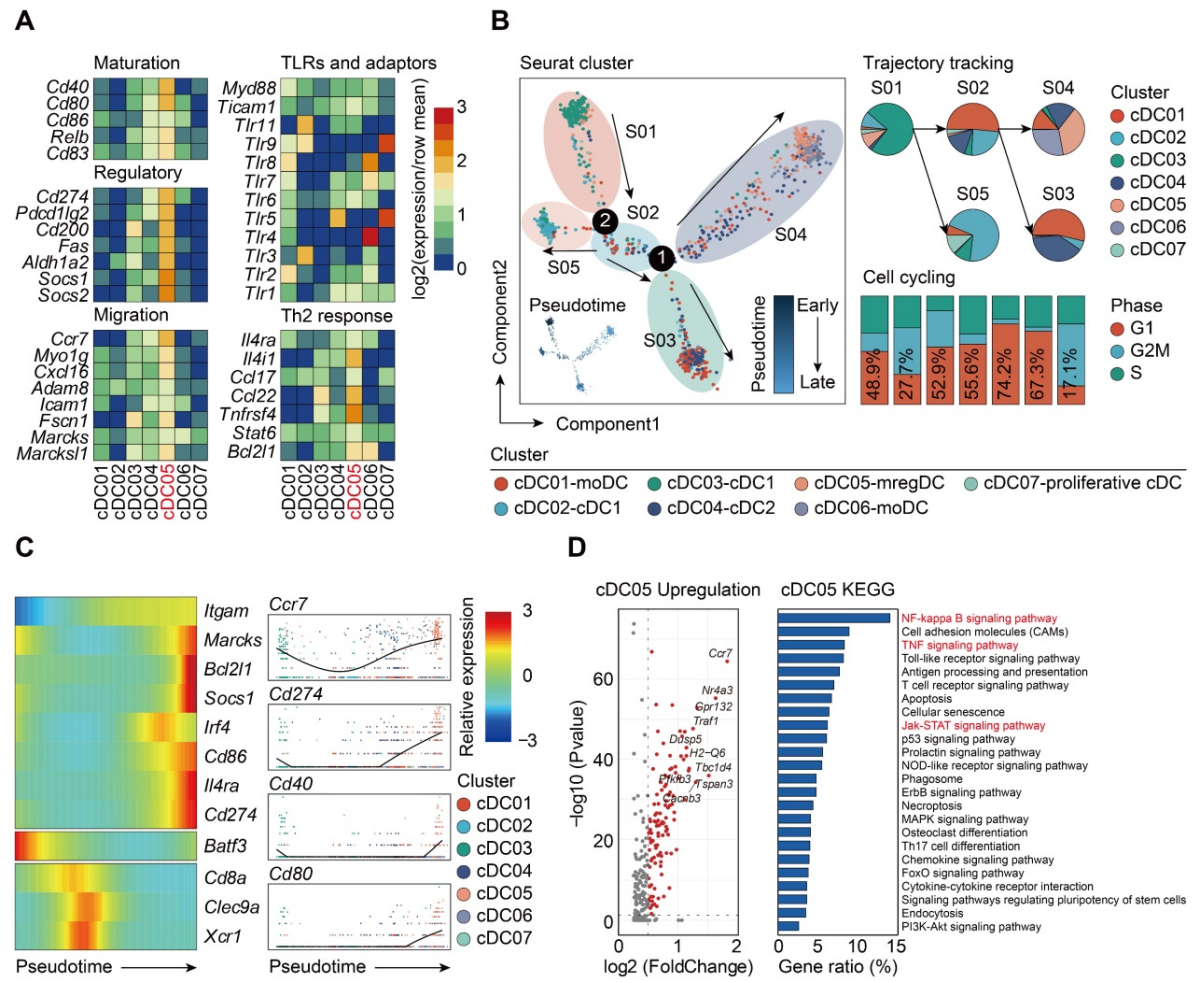
**Sepsis-induced mregDCs exhibit unique transcriptional signatures. A.** Heatmap showing relative expression level of mregDC signature genes among subclustered cDC subsets (blue, low expression level; red, high expression level). **B.** The developmental trajectory of cDCs inferred by Monocle2, colored-coded by the clusters and pseudotime (left panel). Putative trajectory for cell transition states of cDCs, with proportion of each subcluster (upper right panel). Composition of cells at disparate proliferative phases calculated by cell cycling scores (G1, G2M and S) (lower right panel). **C.** Heatmap displaying the dynamic transitions in expression level of cDC1, cDC2 and mregDC marker genes along with the pseudotime (left panel). Pseudotime plots illustrating expression of selected signature genes over pseudotime with distribution of cDC subclusters (right panel). **D.** Volcano plot showing upregulated genes in the cluster of mregDCs (left panel). Bar graph listed the enriched activated pathways in mregDCs by KEGG analysis (right panel).

The specific expression of *Ccr7* led us to question if this type of DCs shared similar features with the recently annotated “mregDCs”, which was characterized in lung cancer as mature DCs enriched in immunoregulatory molecules 38, 67. To test such hypothesis, we assessed the expression of mregDC signature genes among all cDCs clusters identified by our scRNA-seq analyses. Only cDC05 exhibited high levels of maturation-related genes (*Cd40*, *Cd80*, *Relb*, *Cd83*), along with expression of immunoregulatory molecules (*Cd274*, *Pdcd1lg2*, *Cd200*, *Fas*, *Socs1*, *Socs2*, *Aldh1a2*) (Figure 6A). This cluster highly expressed genes associated with cell migration (*Ccr7*, *Myo1g*, *Cxcl16*, *Fscn1*, *Marcks1*) and helper T cell (Th)2 response (*Il4i1*, *Ccl22*, *Tnfrsf4*, *Bcl2l1*), while transcripts for Toll-like receptor (TLR) pathway genes were downregulated. Taken together, our data suggest that the cDC05 cluster closely resembles mregDC, and has feature homologous to *LAMP3^+^* cDCs or *CCR7^+^* cDCs, which have been widely detected in various types of tumors 38, 61, 68-70.

It has been reported that the development of mregDCs might be attributed to both cDC1s and cDC2s differentiation 38. By adopting aforementioned cell cycle and pseudotime analyses, we examined the origin of sepsis-induced mregDCs (Figure 6B; Table S10-S11). The cDC05 cluster associated with mregDCs features was found to be predominantly enriched in state04, which emerged in relatively late phase along pseudotime. This cluster also had the lowest G2M + S score, indicating its diminished proliferative activity. Thus, our data implicated that sepsis-induced mregDCs might be developmentally originated from the other cDCs populations 68. We subsequently investigated the transcriptional alterations correlated with transitional states and found that the signature genes could be further categorized into 3 gene sets (Figure 6C). The first gene set, comprised of mregDC (*Cd86*, *Cd274*, *Marcks*, *Il4ra*) and cDC2 (*Itgam*, *Irf4*) signature genes, was activated at the end of the trajectory. The second and third sets were characterized by cDC1s marker genes (*Batf3*, *Cd8a*, *Clec9a*, *Xcr1*), and they were enriched at the early stage along pseudotime. Pathway analysis of upregulated genes revealed that nuclear factor-kappa B (NF-κB), tumor necrosis factor (TNF), Janus kinase (JAK)-signal transducer and activator of transcription (STAT) signaling pathways were enriched in the cluster of sepsis-induced mregDCs. These signaling pathways play indispensable roles in regulating programmed death-ligand 1 (PD-L1) expression (Figure 6D) 71-73. Therefore, we confirm that cDC05 represents a unique DC cluster induced in sepsis and displays features similar to the previously described “mregDCs” 38, 67. We refer to these cells as mregDCs in the subsequent studies.

### mregDCs exert dual immunoregulatory and immunogenic functions

**Figure 7:**
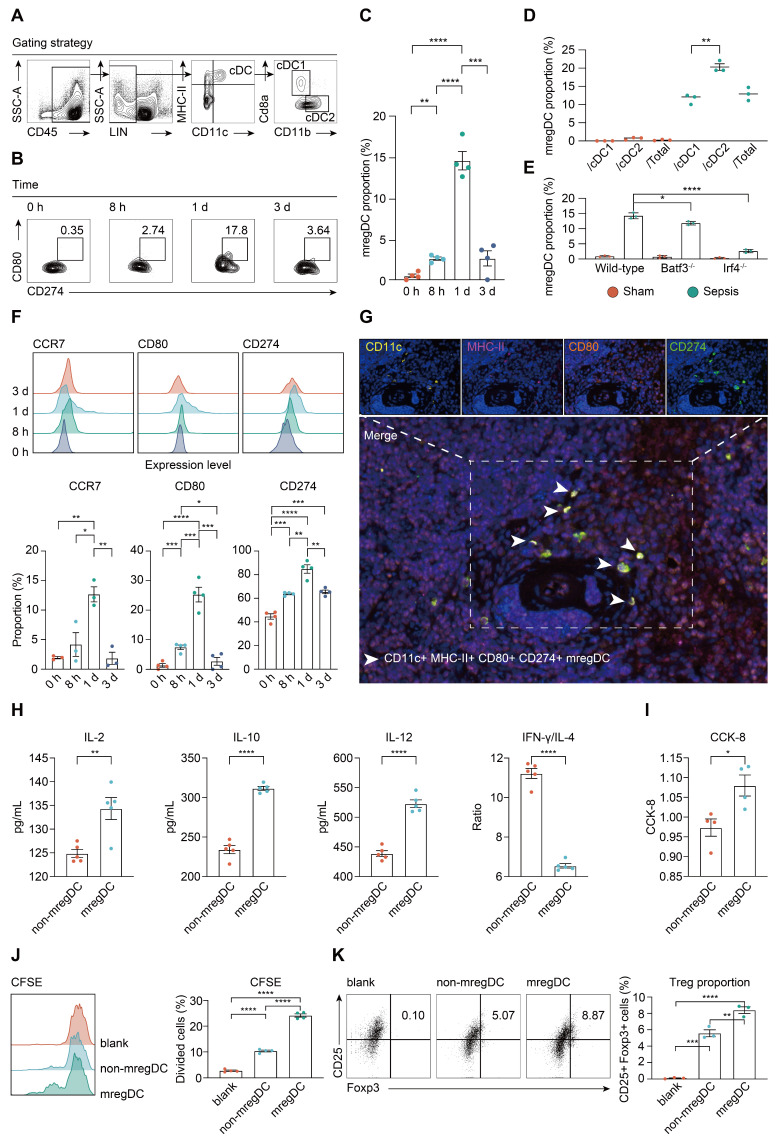
** mregDCs are enriched upon sepsis induction with dual immunoregulatory and immunogenic functions on CD4^+^ T cell responses. A.** Representative contour plots elucidating flow cytometry-based gating strategy for spleen-derived cDCs in wild-type mice. cDCs were defined as CD45^+^ Lin (CD3e, CD19, CD49b, Ly6C)^-^ CD11c^+^ MHC-II^+^ cells, in which cDC1 and cDC2 were defined CD45^+^ Lin^-^ CD11c^+^ MHC-II^+^ CD8a^+^ CD11b^-^ and CD45^+^ Lin^-^ CD11c^+^ MHC-II^+^ CD8a^-^ CD11b^+^ cells, respectively. **B, C.** Frequency of splenic mregDCs as a proportion of total cDCs at disparate time points after CLP operation. mregDCs were defined as CD45^+^ Lin^-^ CD11c^+^ MHC-II^+^ CD274^hi^ CD80^hi^ cells. Contour plots **B.** and quantitative bar charts C. showing alterations in mregDCs proportion upon sepsis induction measured by flow cytometry analysis. **D.** Scatter plot showing mregDCs proportion in cDC1 and cDC2 subsets independently between sham and sepsis groups. **E.** Quantitative bar plots displaying and comparing mregDCs proportion between wild-type, *Batf3*^-/-^ and *Irf4*^-/-^ mice. **F.** Histograms with quantitative bar charts showing proportion of CCR7, CD80, and CD274 positive cells measured by flow cytometry, across distinct time points after onset of sepsis, as a percentage of total cDCs. **G.** Multiplex immunofluorescence images demonstrating the *in situ* existence of mregDCs in spleen after septic challenge, using antibodies, including CD11c, MHC-II, CD80, and CD274. Scale bar, 100 μm. **H.** Quantitative bar charts showing the level of multiple cytokines in supernatants between mregDCs and non-mregDCs groups, including interleukin (IL)-2, IL-4, IL-10, IL-12, and IFN-γ. **I.** Quantitative bar chart displaying the results from cell counting kit-8 (CCK-8) assay. **J.** Histogram with quantitative bar plot illustrating and comparing the proliferative activity of naïve CD4^+^ T cells co-cultured with either mregDCs or non-mregDCs based on carboxyl fluorescein succinyl ester staining (CFSE) assay. **K.** Contour plots with quantitative bar chart showing the proportion of CD4^+^CD25^+^Foxp3^+^ Tregs between mregDCs and non-mregDCs groups. One-way ANOVA with Tukey HSD test **C**, **F**, **J**, **K**; Unpaired two-sided Student's *t* test **D**, **H**, **I**. Data were expressed as means ± SEM. *P<0.05; **P <0.01; ***P<0.001; ****P<0.0001.

We next investigated the change in prevalence of mregDCs during the course of sepsis using flow cytometry analysis. As shown in Figure 7A, CD45^+^ Lin^-^ MHC-II^+^ CD11c^+^ cells were gated from splenic immune cells, in which CD45^+^ Lin^-^ MHC-II^+^ CD11c^+^ CD8a^+^ CD11b^-^ and CD45^+^ Lin^-^ MHC-II^+^ CD11c^+^ CD8a^-^ CD11b^+^ cells were defined as cDC1s and cDC2s, respectively 74. From these cell populations, CD45^+^ Lin^-^ MHC-II^+^ CD11c^+^ CD80^hi^ CD274^hi^ cells were defined as mregDCs based on signature genes of mregDCs. Frequencies of mregDCs as percentages of total DCs were sustainably increased upon induction of sepsis and were highest at 24 h at 15% of total splenic DC, and then decreased at 72 h after CLP (Figure 7B-C). To probe the origins of mregDCs, we analyzed the proportion of mregDCs in cDC1 and cDC2 subsets independently (Figure 7D). mregDCs were more strongly represented with the cDC2 subset. Furthermore, the mregDC module was significantly diminished in *Batf3^-/-^* and *Irf4^-/-^* septic mice in comparison with wild-type mice with sepsis, reduction in *Irf4^-/-^* mice was notably greater (Figure 7E). Although these results suggest that both cDC1 and cDC2 can differentiate into mregDCs, cDC2s derived mregDCs are more abundant compared to those of cDC1s upon septic insults, in agreement with previous work 38, 61, 68. We observed the identical trend regarding the protein expression of mregDC marker genes, including CCR7, CD274 (PD-L1), and CD80, which were consistently upregulated and enriched at 24 h after sepsis (Figure 7F). In addition, we identified MHC-II^+^ CD11c^+^ CD80^+^ CD274^+^ mregDCs in spleens from CLP mice *in stiu* using multiplex immunofluorescence tissue staining (Figure 7G).

To evaluate the unique function of mregDCs on T cell response, splenic mregDCs and non-mregDCs from CLP mice (24 h) were sorted by FACS, and co-cultured with spleen derived CD4^+^ naïve T cells obtained from unmanipulated mice. CD4^+^ T cells co-cultured with mregDCs were more potent in secreting interleukin (IL)-2 and IL-10 compared to those co-cultured with non-mregDCs (Figure 7H). mregDCs were also found to upregulate IL-12 production in comparison to those of non-mregDCs. The ratio of interferon (IFN)-γ to IL-4 was significantly lower in the supernatants obtained from mregDCs co-cultured with CD4^+^ T cells, indicating a phenotypic shift to Th2 cells, in agreement with the mregDC gene module (Figure 6A). Using both cell counting kit-8 (CCK8) and carboxyl fluorescein succinyl ester staining (CFSE) assays, we found that mregDCs possessed more potent than non-mregDC in driving the activation/ proliferation of CD4^+^ naïve T cells *in vitro* (Figure 7I-J). Similarly, mregDCs were more effective in potentiating the differentiation of naïve T cells toward CD4^+^ CD25^+^ Foxp3^+^ Treg than non-mregDCs (Figure 7K) 38. Thus, sepsis-induced mregDCs might exert dual immunoregulatory and immune stimulatory functions on CD4^+^ T cell responses.

### NF-κB and JAK-STAT pathways are critically involved in mediating the mregDC program

**Figure 8:**
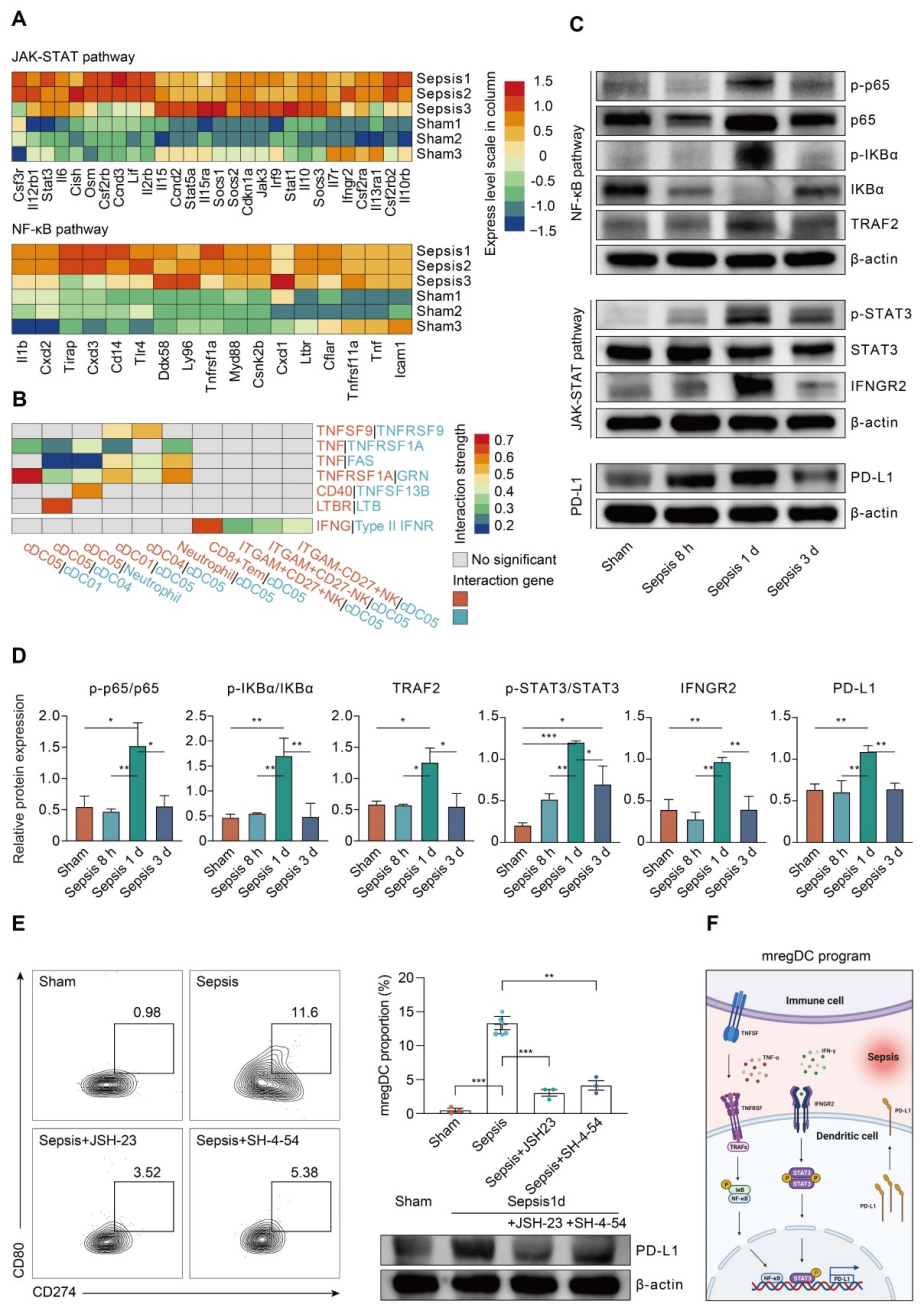
** Sepsis-associated mregDC program is initiated in a TNFRSF-NF-κB and IFNGR2-STAT3 dependent manner. A.** Histogram indicating the relative expression level of selected genes related to JAK-STAT and NF-κB pathways from bulk RNA-seq analyses (blue, low expression level; red, high expression level). **B.** Histogram showing the cell-cell communication between mregDCs and other immune cell types, based on selected ligand-receptor pairs in association with TNFRSF-TNFSF and IFN-γ- IFNGR2. Statistical significance (*P*<0.05) was determined by permutation test from CellPhoneDB, with color of grey indicating no statistical significance. The color gradient indicated the level of interaction (blue, low level of interaction; red, high level of interaction). **C.** Representative Western blotting images of splenic CD11c^+^ DCs isolated from wild-type mice undergone sham or CLP operation at distinct time points. **D.** Quantitative bar charts displaying the results of Western blotting analyses. The values represent protein levels relative to the unphosphorylated form or β-actin level. The data shown are representative of 3 independent experiments. **E.** Inhibitory experiments were performed to validate the effect of TNFRSF-NF-κB and IFNGR2-STAT3 pathways on mregDC program using STAT3 and NK-κB inhibitors, JSH-23 and SH-4-54, respectively. Contour plots (left panel) with quantitative bar chart (upper right panel) showing the proportion of mregDCs upon inhibition of STAT3 and NK-κB. Western blot analysis of PD-L1 expression in splenic CD11c^+^ DCs after treatment with inhibitors (lower right panel). **F.** Proposed model of mregDC program upon sepsis induction. Graphs were created with BioRender.com. Statistical significance was calculated using one-way ANOVA with Tukey HSD test **D**, **E**. Data were expressed as means ± SEM. *P<0.05; **P<0.01; ***P<0.001.

To clarify the regulators of the mregDC program upon sepsis induction, we explored the contribution of pathways known to mediate expression of PD-L1 71. Corresponding to the KEGG analysis, NF-κB and JAK-STAT signaling pathways are known to activate the mregDC program (Figure 6D). The roles of their downstream transcriptional factors, STAT3 and RELA (p65, a subunit of NF-κB) in regulating PD-L1 expression have been well established, prompting us to probe their potential impacts on mregDC differentiation in the setting of sepsis 71, 75, 76. By integrating splenic CD11c^+^ cDCs bulk-seq data, we noted a substantial transcriptional activation of gene sets associated with both NF-κB and JAK-STAT pathways at 24 h after CLP surgery, including *Jak3*, *Stat1/3/5a*, *Socs1/2/3*, *Tirap*, and *Myd88* (Figure 8A). We further investigated the possible receptors and molecular axis downstream that potentiated NF-κB and JAK-STAT signals, thereby mediating upregulation of PD-L1. As we known, IFN-γ receptor (IFNGR) and TNF receptor superfamily (TNFRSF) signaling are a well-studied molecular axis in mediating STAT3- and p65-dependant PD-L1 expression 73, 77, 78. In the present experiments, genes encoding IFNGR (*Ifngr2*) and TNFRSF proteins (*Tnfrsf1a* and *Tnfrsf11a*) were found to be upregulated in the CLP group compared to the sham group, urging us to hypothesize the existence of interacting receptor-ligand pairs with respect to TNFRSF and IFNGR2 between mregDCs and other immune cell types (Figure 8A-B). Interestingly, using cell-cell communication analysis, mregDCs were noticed to express high levels of TNFRSF receptors *TNFRSF9* (*4-1BB*), *TNFRSF1A* (*TNFAR*), *FAS*, *CD40*, and *LTBR*. They interacted with their cognate ligands expressed on moDCs, cDC2s as well as neutrophils, including *TNF*, *TNFSF9* (*4-1BBL*), *GRN*, *TNFSF13B* (*BAFF*), and *LTB*. In addition, CD8^+^ Tem and various NK subsets putatively released IFN-γ, thereby signaling to the IFNGR2 on mregDCs.

We further validated these findings in the protein level. A time course study using splenic CD11c^+^ DCs isolated from CLP mice revealed that the phosphorylation of p65 and IκBα was highest at 24 h after sepsis, in parallel with the activation of TNF receptor-associated factor 2 (TRAF2), which served as a pivotal adaptor in transmitting signals from TNFRSF (Figure 8C-D). Likewise, phosphorylation of STAT3 was markedly augmented at 24 h after the onset of sepsis, in combination with simultaneous induction of IFNGR2. Correspondingly, sepsis induced PD-L1 expression on DCs increases at 24 h and then markedly diminished at 72 h after CLP surgery. Meanwhile, the elevated proportion of mregDCs as well as PD-L1 expression levels in sepsis were significantly attenuated following inhibition of p65 and STAT3 *via* specific inhibitor JSH-23 as well as SH-4-54, respectively (Figure 8E). As shown in Figure 8F, these results implicate a model in which sepsis-induced mregDCs program is developmentally dependent on both TNFRSF-NF-κB and IFNGR2-STAT3 axes.

### Validating our findings in multiple organs and human sepsis

**Figure 9:**
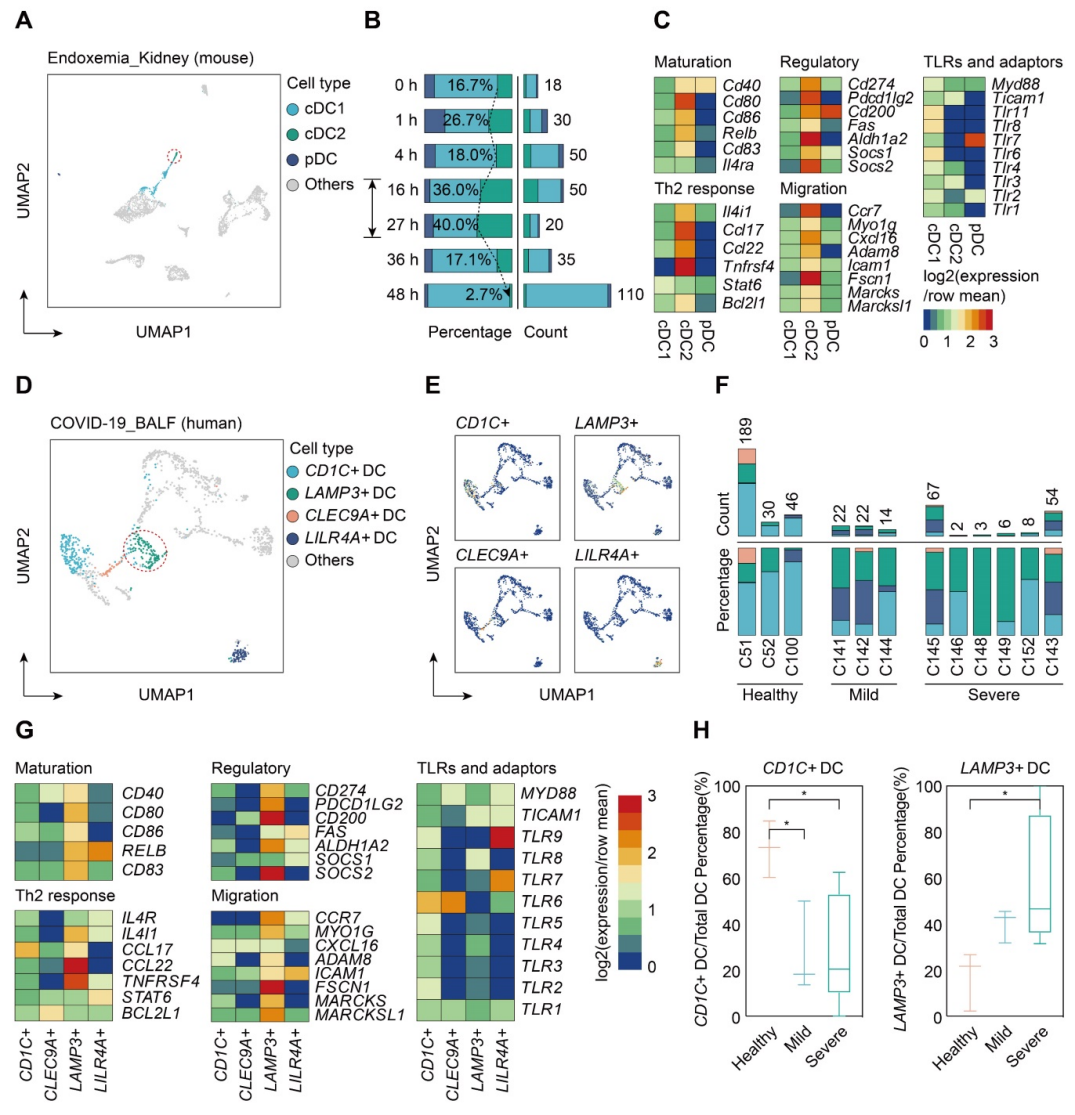
** Single-cell analyses of publicly available data demonstrate multiorgan and cross-species conservation of sepsis-induced upregulation of mregDCs. A.** Single-cell analysis of previously published scRNA-seq dataset containing various immune cell subpopulations in lipopolysaccharide (LPS) challenged murine kidney projected in UMAP, with color-coded DC subtypes, including cDC1, cDC2, and plasmacytoid dendritic cell (pDC). **B.** Proportion and absolute counts of renal cDC2s as a percentage of total DCs, across distinct time points after the onset of endoxemia. **C.** Heatmap showing relative expression level of mregDC signature genes among subclustered DC subsets (blue, low expression level; red, high expression level). **D.** Unbiased Seurat-based clustering analysis of immune cells derived from bronchoalveolar lavage fluid (BALF) of the novel coronavirus disease (COVID-19) patients yielded four disparate clusters of DCs visualized by UMAP. **E.** Phenotypic annotations of each DC subcluster were presented in independent UMAP plots based on the relative expression level of cell-type specific genes, including *CD1C*, *CLEC9A*, *LAMP3*, and* LILR4A* (blue, low expression; yellow, high expression). **F.** Histogram indicating proportion and absolute counts of each DC subpopulation in BALF from healthy individuals and COVID-19 patients with disparate severity, as a percentage of total DCs. **G.** Heatmap displaying expression level of selected mregDC marker genes for each DC subset (blue, low expression level; red, high expression level). **H.** Quantitative box plots showing and comparing the proportion of *CD1C^+^* DCs (left panel) and *LAMP3^+^* DCs (right panel) among healthy participants and mild or severe cases of COVID-19. Statistical significance was determined using one-way ANOVA with Tukey HSD test **H**. Data were presented as means ± SEM. *P<0.05.

To determine whether sepsis-induced mregDCs were presented in other tissues in the setting of sepsis, we conducted a secondary analysis of scRNA-seq data from a study that comprehensively profiled the spatial and temporal transcriptomic alterations of kidneys in a murine lipopolysaccharide (LPS) model 45. We reanalyzed the data using an unsupervised UMAP clustering method that focused on the DC subsets in line with our definitions (Figure 9A; Table S12). The identified DC population could be further categorized into three subclusters based on expression level of canonical genes, including cDC1s, cDC2s, and pDCs. By comparing the temporal change in each cluster after LPS administration, cells form cDC2s were preferentially enriched at 16 and 27 h after LPS stimulation, implying that the proportion of these cell types might reach the peak between 16 and 27 h after LPS challenge (Figure 9B). In support of our CLP results in spleen, we clarified a kidney-resident cDC2s-like cluster that highly shared characteristics of mregDCs (Figure 9C; Table S13). Therefore, we showed the evidence for the presence of cDC2s originated mregDCs in murine kidneys during endotoxemia, an acute insult with characteristics similar to sepsis.

Lastly, we sought to determine whether cells with features of mregDC could be found in human sepsis. Up to now, four scRNA-seq based studies focusing on human sepsis have been reported, all using only PBMCs 26-28, 79. By reanalyzing the data from the study by Reyes et al., we found no evidence of a mregDC cluster in the circulating DC cell populations, in accordance with the tissue-resident feature of mregDCs (Figure S5A-B). Patients hospitalized with COVID-19 are at high risk of developing severe acute respiratory syndrome associated sepsis, especially in severe cases 80-82. Therefore, we reanalyzed scRNA-seq data from a study that included cells from bronchoalveolar lavage fluid (BALF), sputum, and pleural fluid 39. Re-clustering of cells from BALF samples revealed four DC subclusters based on expression of marker genes *CD1C^+^* DCs, *CLEC9A^+^* DCs, *LAMP3^+^* DCs, and* LILR4A^+^* DCs (Figure 9D-F; Table S12). *CD1C^+^* DCs were characterized by high expression of *CD1C* and *FCER1A*, thus representing cDC2s, whereas *CLEC9A^+^* DCs specifically expressed *CLEC9A* and *XCR1*, corresponding to cDC1s. Meanwhile, *LILR4A^+^* DCs were annotated as pDCs. Similar to our results in the experimental model, *LAMP3^+^* DCs in COVID-19 patients were identified as a cluster of human sepsis associated mregDCs that highly expressed maturation (*CD40*, *CD80*, *CD86*, *CD83*)- and migratory (*CCR7*, *MYO1G*, *ADAM8*, *FSCN1*, *MARCKS*, *MARCKSL1*)-related marker genes as well as immunoregulatory molecules (*CD274*, *PDCD1LG2*, *CD200*, *ALDH1A2*, *SOCS2*), along with low expression of TLR signaling pathway genes (Figure 9G; Table S13). Strikingly, a significantly elevated percentage of *LAMP3^+^* DCs could be observed in the BALF of six severe COVID-19 cases in comparison to cells from healthy controls or mild to moderate COVID-19 patients. Furthermore, the proportion of *CD1C^+^* DCs was markedly higher in healthy controls compared to COVID-19 patients, suggesting a positive correlation between elevation of mregDCs and the progression of SARS-CoV-2 infection-induced sepsis (Figure 9H; Table S14).

## Discussion

This landscape study of the single cell transcriptomic changes in sepsis demonstrates the remarkable heterogeneity within the immune cell populations in response to severe sepsis across tissues and time. Among the many notable findings, we unexpectedly notice that sepsis induces marked heterogeneity within cDC that includes the appearance of mregDC in multiple organs. We confirm that these cells simultaneously exert profound immune stimulatory and regulatory changes in CD4^+^ T cells. The transcriptome profiling in combination with trajectory and cell-cell interaction network analyses provides a comprehensive and dynamic picture for understanding how immune cells within different tissues differentiate and crosstalk with each other. The novel findings along with the foundational datasets will serve as a valuable resource to guide future research in critical illness.

The mregDC-like cells we identified in sepsis closely resembled the mregDCs recently described within human cancers 38, 68. Intriguingly, in contrast to the previous study where tumor-associated mregDCs lacked expression of cDC1 and cDC2-related signature genes, we noted that sepsis-induced mregDCs expressed low or intermediate level of cDC2-specific markers, including *Sirpa*, *Runx3*, and *Zbtb46*. Meanwhile, the UMAP clustering analysis closely associated sepsis mregDCs with the canonical cDC2 subset, suggesting that sepsis-induced mregDCs might originate from cDC2s. Nevertheless, the expression pattern of gene sets related to cDC1s, cDC2s, and mregDCs along with pseudotime analysis elucidated a transition from cDC1s to mregDCs, as shown by the upregulation of mregDC signature genes in the very late phase of cell differentiation and away from the expression of cDC1-specific genes, such as *Batf3*, *Cd8a*, *Clec9a*, and *Xcr1*, implying that cDC1s could also differentiate to mregDCs. At the protein level, by gating mregDCs in cDC1s and cDC2s independently, we demonstrated that both lineages could become the source of mregDCs 38, 61, 68. Furthermore, we found a statistically significant reduction in mregDCs proportion upon deletion of *Batf3* and *Irf4*, leading to the specific loss of cDC1s and cDC2s, respectively. Thus, mregDCs likely originate from both cDC1s and cDC2s, while cDC2-derived mregDCs are predominantly enriched in the setting of sepsis.

A previous study on non-small-cell lung cancer suggested that induction of the mregDC program in cDC1s could be attributed to the uptake of tumor cell-associated antigen. In this report, antigen uptake of cDC1s was specifically dependent on the phagocytic cell-surface receptor AXL in association with the load of apoptotic cells within tumor microenvironment 38. Herein, our scRNA-seq data showed no upregulated expression of phagocytic cell-surface receptors in the cluster of sepsis-associated mregDCs. Instead, cell-cell communication analysis identified enriched receptor-ligand interactions between mregDCs and other immune cell types *via* TNRSF/TNFSF/TNF-α and IFNGR2/IFN-γ. Our findings point to the TNFRSF-NF-κB and IFNGR2-JAK-STAT3 signaling axes as the main signaling pathways potentiating mregDC program. These results were further validated in the protein level, along with inhibitory experiments, indicating the critical role of two TFs, NF-κB and STAT3, in modulating sepsis-induced PD-L1 upregulation in DCs. Consistent with our hypothesis, a previous report demonstrated that *in vitro* stimulation of LPS + IFN-γ facilitated the differentiation of naïve primary cDC1, cDC2, and pDCs toward *LAMP3*^+^ DCs, representing a subset that was homogenous to mregDCs in homo sapiens 61. It was notable that various inflammatory mediators peaked at 24 h and were then substantially diminished by 72 h after CLP, corresponding with the prevalence of mregDCs. Therefore, it appears that the priming of the sepsis-associated mregDC program is closely correlated with the hyperinflammatory stage during sepsis.

Based on the functional analyses, we found that sepsis-induced mregDCs were potent at driving the phenotypic shift of naïve CD4^+^ T cells towards Tregs and Th2, implicating a role for mregDCs in promoting immune suppression. This would be in accordance with prior pan-cancer analyses on cDC2-derived *LAMP3*^+^ cDCs 68, 72. Given the important role of Treg and Th2 in promoting tissue repair, it could be that mregDCs were induced to counter sepsis-related hyperinflammation and to alleviate organ damage. However, at the same time mregDCs were capable of activating CD4^+^ T cells *in vitro*, as shown by elevated levels of IL-2 and IL-12, and enhanced proliferation. Hence, sepsis-associated mregDCs might exert diverse functions with high immunogenicity, consistent with a recent study that proposed dual functions for cDC1-derived mregDCs in upregulating Treg and CD8^+^ T cells 38. Altogether, this study uncovers an underlying mechanism, during which the mregDC module is actively initiated upon sepsis-induced hyperinflammation and these cells migrate to multiple organs. We speculate that within organs mregDC can ameliorate organ dysfunction and promote tissue repair *via* restoring homeostasis of the immune microenvironment.

Several limitations should be taken into consideration when interpreting our findings. Firstly, the precise mechanisms underlying the regulation of mregDC program during sepsis require further investigation. While we demonstrate roles for NF-κB and STAT3 in driving differentiation toward mregDCs, further analysis including an assessment of open chromatin patterns in cDC should be performed to fully decipher the transcriptional regulators and ontogeny of this subset 28. Secondly, receptors involved in the induction of mregDCs program remain largely elusive. Although we demonstrate that hyperinflammation could be one of the prerequisites for the development of mregDCs, the stimuli and receptors-ligand pairs have not been fully interrogated. Given the complexity of the pathogenesis of sepsis, further well-designed *in vitro* experiments using single stimuli are needed to validate the findings from cell-cell interacting analyses. Finally, it is not yet feasible to selectively delete mregDC to fully assess the roles of these cells in the immune response to septic challenge.

## Conclusions

In summary, we have generated a comprehensive single-cell immune landscape for polymicrobial sepsis, in which we identify the significant alterations and heterogeneity in immune cell subsets that take place during sepsis. More importantly, we find a conserved and potentially targetable immunoregulatory program within DCs that associates with hyperinflammation and organ dysfunction early following sepsis induction. Our results generalize prior reports that deciphered mregDC program in multiple tumor types to demonstrate the mregDC program in critical illness. This example indicates that this dataset can serve as a rich resource to gain insight into the cellular and molecular basis of sepsis pathogenesis, thereby contributing to the identification of novel biomarkers and therapeutic targets modulating host immune response in sepsis.

## Supplementary Material

Supplementary figures.

Supplementary table.

## Figures and Tables

**Table 1:** Criteria for the histological scoring of organ injuries

Organ injuries	Items
Pulmonary injury	Pulmonary edema
	Parenchymal congestion
	Alveolar hemorrhage
	Peribronchial inflammation
	Perivascular inflammation
	Interstitial inflammation
Liver injury	Ischemic necrosis
	Parenchymal congestion
	Hepatocellular injury
	Periportal inflammation
	Vacuolary degeneration
Renal injury	Congestion
	Glomerular necrosis
	Tubular necrosis
Cardiac injury	Parenchymal congestion
	Necrosis
	Inflammation

Scores for each criterion were given as: 0, none; 1, mild; 2, moderate; 3, severe. At least three microscopic areas were evaluated for each slide.

## Abbreviations

scRNA-seq: single-cell RNA sequencing
CLP: cecal ligation and puncture
cDCs: conventional dendritic cells
ICUs: intensive care units
PBMCs: peripheral blood mononuclear cells
APCs: antigen-presenting cells
MHC-II: major histocompatibility complex class II
moDCs: monocyte-derived DCs
NK cells: natural killer cells
COVID-19: coronavirus disease 2019
FACS: fluorescence-activated cell sorting
DNT: double negative T cells
Treg: regulatory T cells
Tcm: central memory T cells
Tem: effector memory T cells
MZB: marginal zone B
MPP: multipotent progenitor
MEP: megakaryocyte-erythroid progenitor
GMP: granulocyte-monocyte progenitor
CLP: common lymphoid progenitor
TFs: transcription factors
IRF8: interferon regulatory factor 8
TLR: Toll-like receptor
NF-κB: nuclear factor-kappa B
TNF: tumor necrosis factor
JAK: Janus kinase
STAT: signal transducer and activator of transcription
PD-L1: programmed death-ligand 1
IL-2: interleukin-2
IFN-γ: interferon-γ
CCK8: cell counting kit-8
CFSE: carboxyl fluorescein succinyl ester staining
IFNGR: IFN-γ receptor
TNFRSF: TNF receptor superfamily
TRAF2: TNF receptor-associated factor 2
LPS: lipopolysaccharide
BALF: bronchoalveolar lavage fluid
