# Supplementary material for: Single-cell transcriptome profiling of the immune space-time landscape reveals dendritic cell regulatory program in polymicrobial sepsis
Source: Theranostics. 2022 May 29;12(10):4606–28. doi: 10.7150/thno.72760 (PMC9254255; doi:10.7150/thno.72760)
Supplement: Supplementary file 1 — Supplementary figures. [file thnov12p4606s1.pdf]

Supplementary Figures

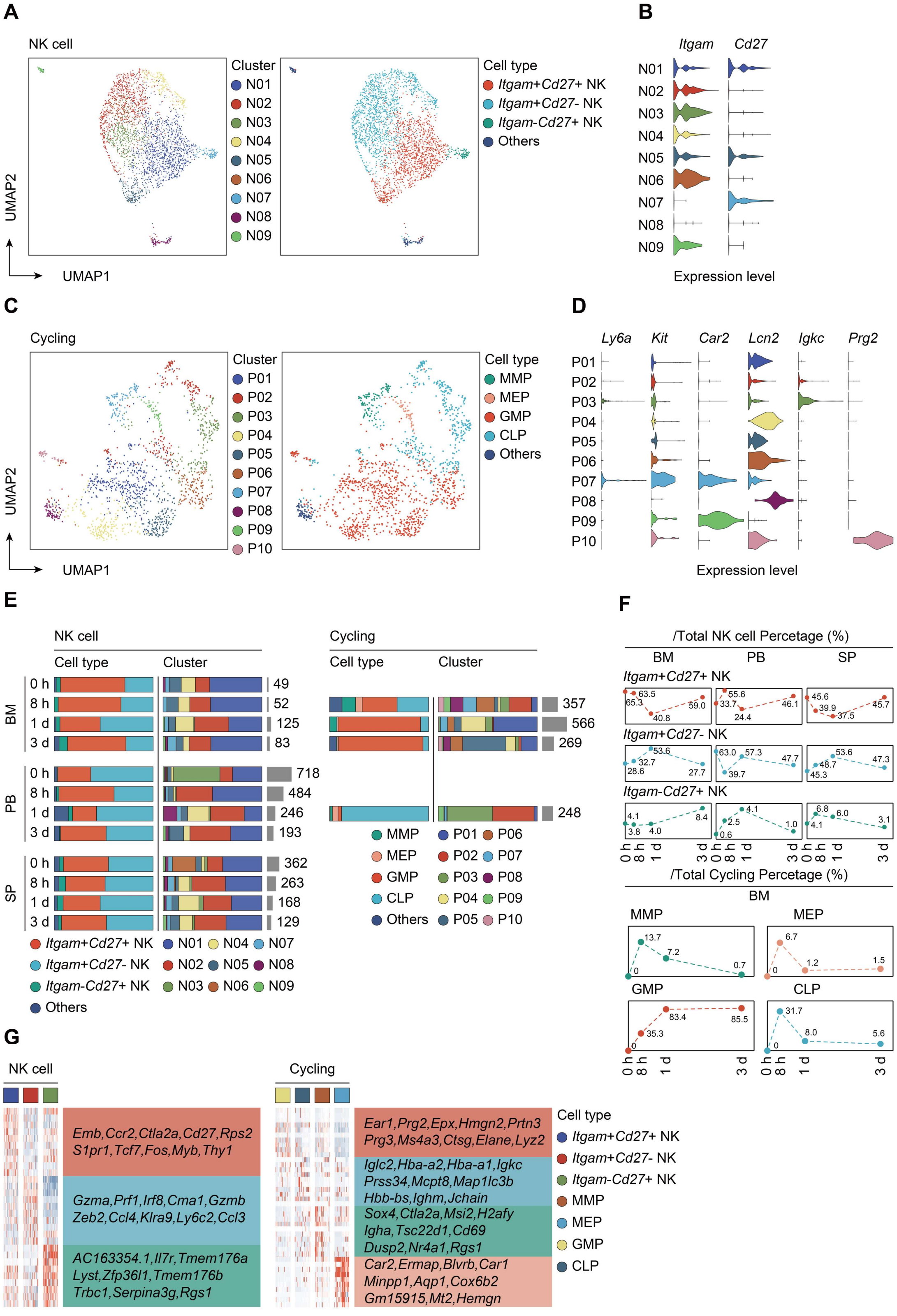

Figure S1. Subtype analyses of NK and cycling cells based on single-cell gene expression. A. UMAP plot of subclustered natural killer (NK) cells, labeled in different colors. Annotations of each subcluster were presented in independent UMAP projection, including 2 *Itgam*$^{+}$ *Cd27*$^{+}$ NK cell subsets, 5 *Itgam*$^{+}$ *Cd27*$^{-}$ NK cell subsets, and 1 *Itgam*$^{-}$ *Cd27*$^{+}$ NK cell subset. **B.** Violin plots showing expression level of selected marker genes for annotation. **C.** Subclusters of cycling cells were visualized and color-coded by UMAP projection. Annotations of each subcluster were summarized in independent UMAP plots, including 1 multipotent progenitor (MPP) subcluster, 1 megakaryocyte-erythroid progenitor (MEP) subcluster, 5 granulocyte-monocyte progenitor (GMP) subclusters, and 2 common lymphoid progenitor (CLP) subclusters. **D.** Violin plots indicating expression level of selected gene signatures used for annotation. **E.** Proportion and absolute counts of each subclustered NK and cycling cells, across disparate immune-relevant tissue sites and distinct time points after CLP. **F.** Percentage of each identified NK/cycling cells subpopulations at distinct time points across all tissue sites. **G.** Heatmaps showing the expression level of top 10 cell type-specific genes among NK and cycling cell subtypes.

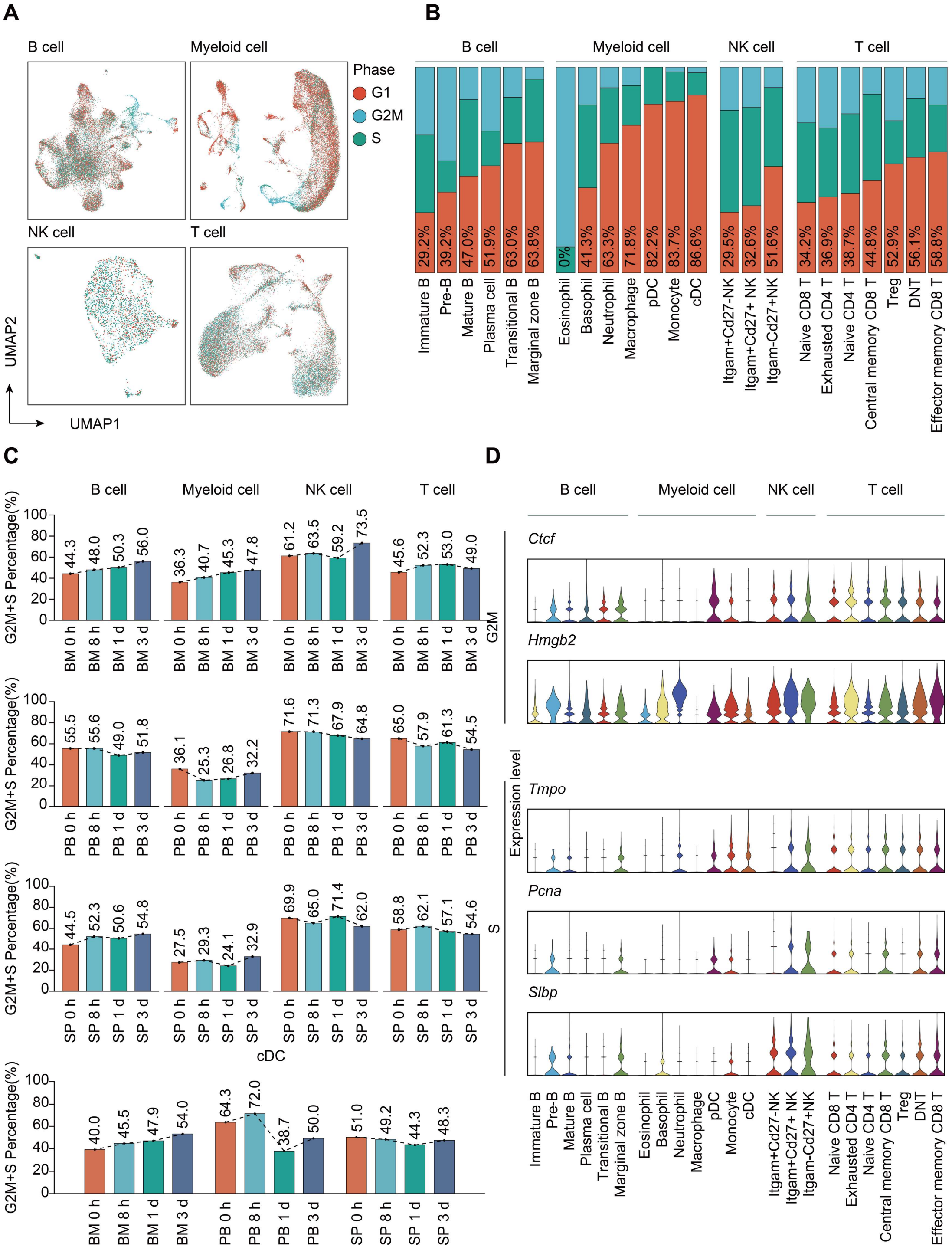

Figure S2. Cell cycle analyses on major immune cell populations. A. UMAPs showing major immune cell populations color-coded by disparate proliferative phases (G1, G2M or S phase), based on cell cycle score assigned for each single-cell. **B.** proportion of cells

at disparate phases for each subclustered immune cell type. **C.** Proportion of cells at G2M + S phase as a percentage of total cells, across different immune-relevant tissue sites and distinct time points after CLP operation. **D.** Violin plots indicating the relative expression level of selected genes for calculating cell cycle score.

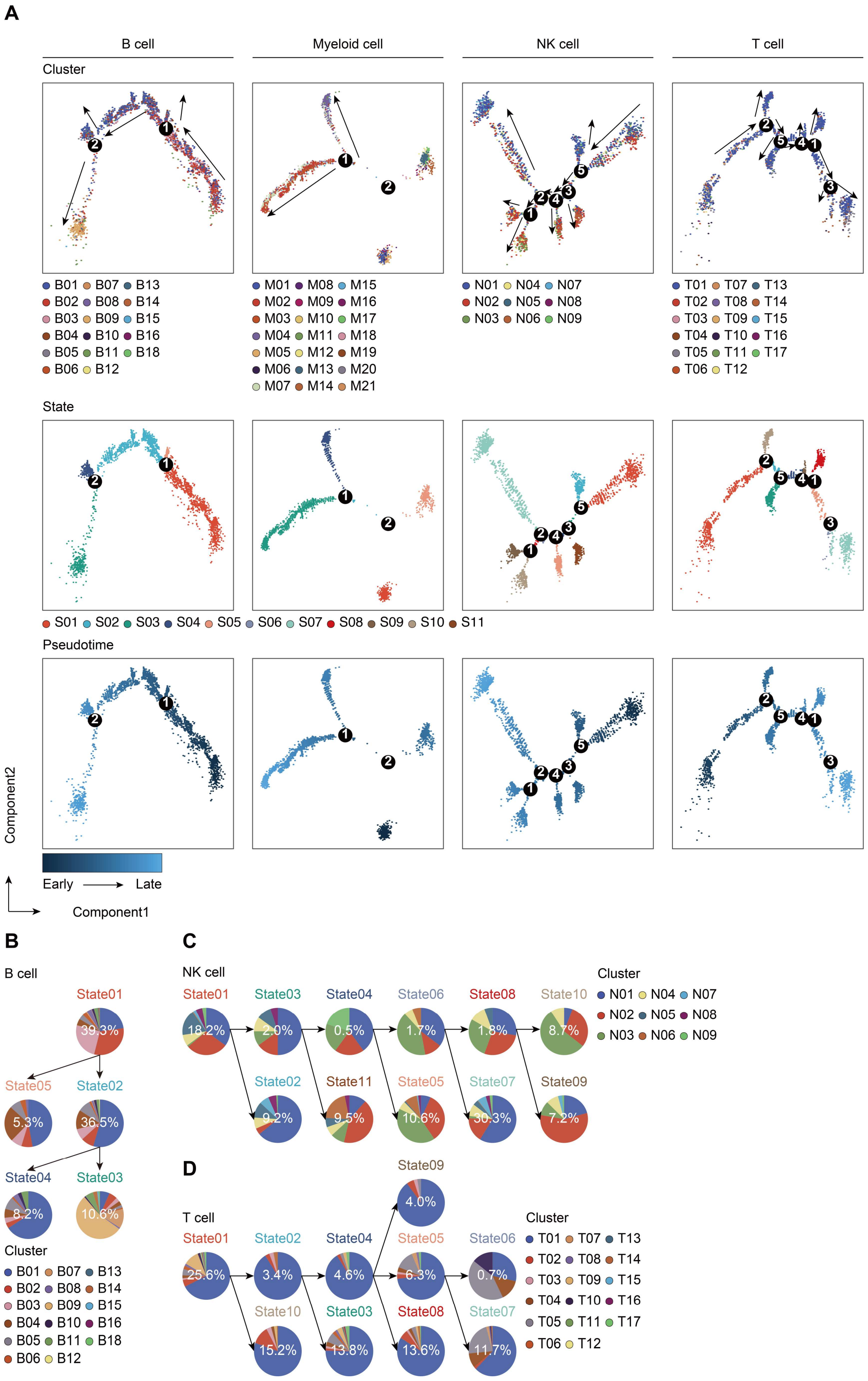
A
B cell
Myeloid cell
NK cell
T cell
Cluster
B01 B07 B13
B02 B08 B14
B03 B09 B15
B04 B10 B16
B05 B11 B18
B06 B12
M01 M08 M15
M02 M09 M16
M03 M10 M17
M04 M11 M18
M05 M12 M19
M06 M13 M20
M07 M14 M21
N01 N04 N07
N02 N05 N08
N03 N06 N09
T01 T07 T13
T02 T08 T14
T03 T09 T15
T04 T10 T16
T05 T11 T17
T06 T12
State
S01 S02 S03 S04 S05 S06 S07 S08 S09 S10 S11
Pseudotime
Component2
Early
Late
Component1
B
B cell
State01
39.3%
State05
5.3%
State02
36.5%
State04
8.2%
State03
10.6%
Cluster
B01 B07 B13
B02 B08 B14
B03 B09 B15
B04 B10 B16
B05 B11 B18
B06 B12
C
NK cell
State01
18.2%
State03
2.0%
State04
0.5%
State06
1.7%
State08
1.8%
State10
8.7%
State02
9.2%
State11
9.5%
State05
10.6%
State07
30.3%
State09
7.2%
Cluster
N01 N04 N07
N02 N05 N08
N03 N06 N09
D
T cell
State09
4.0%
State01
25.6%
State02
3.4%
State04
4.6%
State05
6.3%
State06
0.7%
State10
15.2%
State03
13.8%
State08
13.6%
State07
11.7%
Cluster
T01 T07 T13
T02 T08 T14
T03 T09 T15
T04 T10 T16
T05 T11 T17
T06 T12

Figure S3. Developmental trajectory analyses on major immune cell populations. A. UMAP plots showing the developmental trajectories of major immune cell groups inferred by Monocle2, colored-coded by the clusters (upper panel), stages (middle panel), and pseudotime (lower panel). **B.** Putative trajectory for cell states of transition of B cells with proportion of each subcluster. **C.** Putative trajectory for cell states of transition of NK cells with proportion of each subcluster. **D.** Putative trajectory for cell states of transition of T cells with proportion of each subcluster.

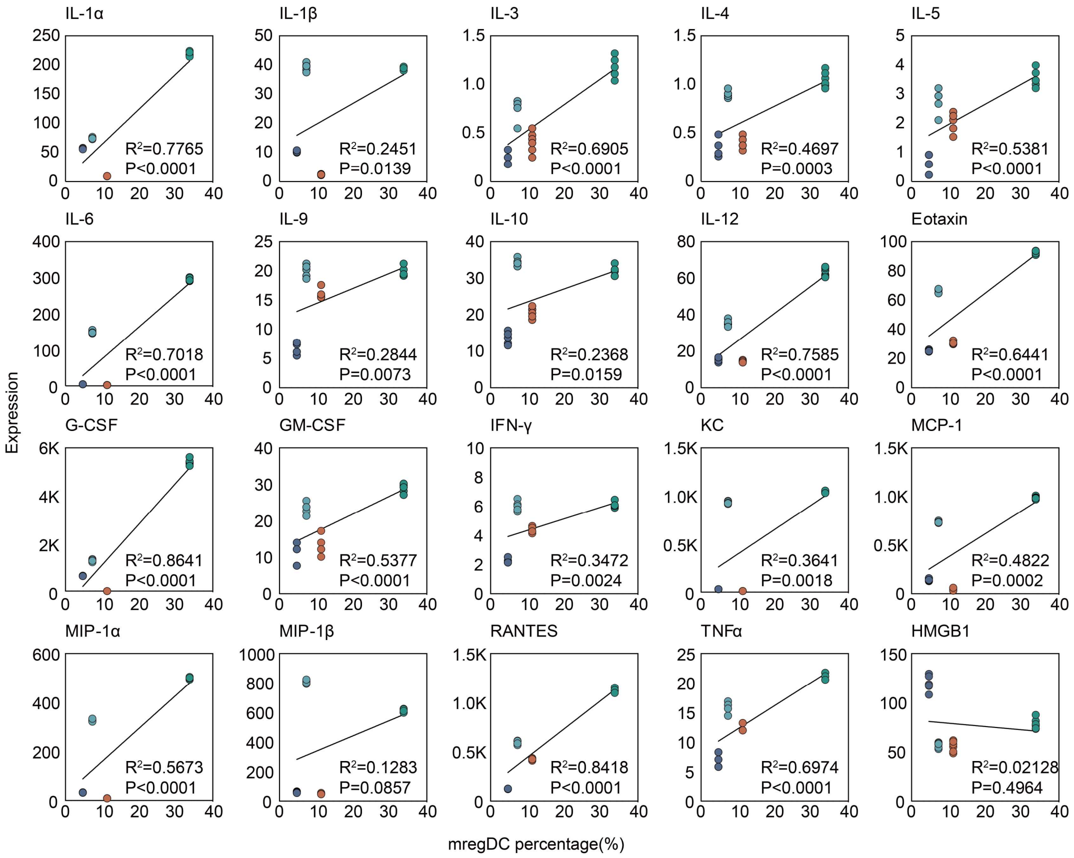

Figure S4. Linear regression analyses on association between mregDC percentage and expression levels of multiple inflammatory cytokines. The linear regression models were constructed to determine the correlation between splenic mregDCs ratio and expression levels of each cytokine or chemokine in mouse spleen, as visualized by fitted linear function. R^2 represents the goodness of fit and a two-tailed $P < 0.05$ is considered as having statistical significance.

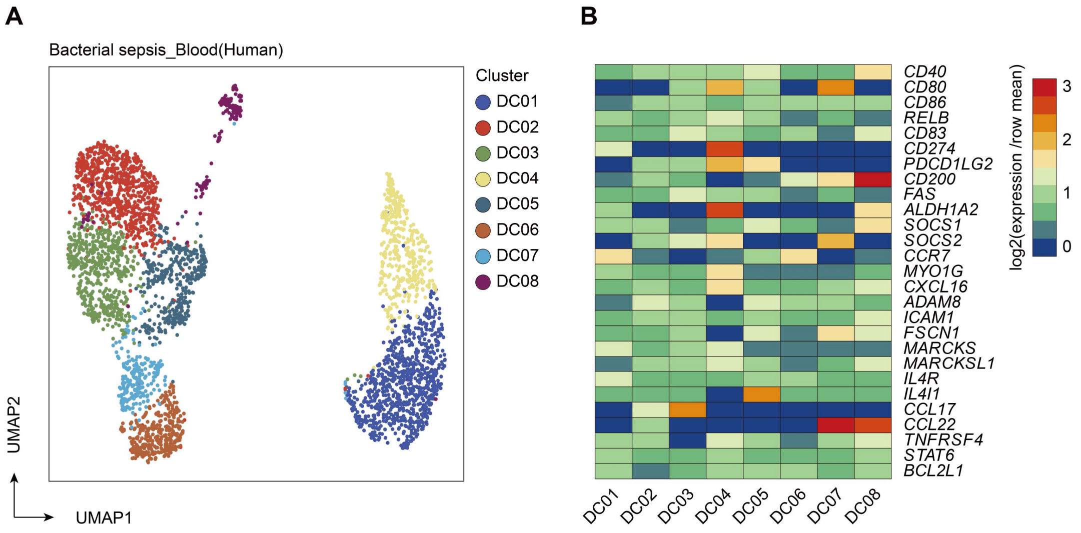

Figure S5. Single-cell analysis of peripheral blood mononuclear cells from patients with bacterial sepsis. A. Re-clustering of dendritic cells derived from peripheral blood mononuclear cells (PBMCs) of patients with bacterial sepsis yielded eight disparate clusters of DCs visualized by UMAP. **B.** Heatmap showing relative expression level of mregDC signature genes among subclustered DC subsets (blue, low expression level; red, high expression level).
